# Determining the microbial and chemical contamination in Ecuador’s main rivers

**DOI:** 10.1038/s41598-021-96926-z

**Published:** 2021-09-03

**Authors:** Dayana Vinueza, Valeria Ochoa-Herrera, Laurence Maurice, Esteban Tamayo, Lorena Mejía, Eduardo Tejera, António Machado

**Affiliations:** 1 Instituto de Microbiología, Colegio de Ciencias Biológicas y Ambientales (COCIBA), Universidad San Francisco de Quito (USFQ), Diego de Robles y Vía Interoceánica, Campus Cumbayá, Casilla Postal 17-1200-841, Quito, 170901 Ecuador; 2 Colegio de Ciencias e Ingeniería, Instituto Biósfera, Universidad San Francisco de Quito (USFQ), El Politécnico, Quito, 170901 Ecuador; 3 Department of Environmental Sciences and Engineering, Gillings School of Global Public Health, University of North Carolina at Chapel Hill, Chapel Hill, NC 27599 USA; 4 Geosciences Environnement Toulouse, CNRS/IRD/CNES/Université Paul Sabatier, 14 avenue Edouard Belin, 31400 Toulouse, France; 5 Área de Salud de la Universidad Andina Simón Bolívar, Área de Salud, Toledo N22-80, P.O. Box 17-12-569, Quito, 170143 Ecuador; 6 Facultad de Ingeniería y Ciencias Agropecuarias Aplicadas, Grupo de Bioquimioinformática, Universidad de Las Américas, Quito, 170125 Ecuador

**Keywords:** Environmental chemistry, Environmental impact, Environmental microbiology, Policy and public health in microbiology

## Abstract

One major health issue is the microbial and chemical contamination of natural freshwater, particularly in Latin American countries, such as Ecuador, where it is still lacking wastewater treatment plants. This study analyzed the water quality in twelve rivers of Ecuador (Coastal, Andean, and Amazonian regions). All rivers showed levels of *E. coli* and total coliforms above the maximum limit according to International and Ecuadorian legislations. The most polluted rivers were Zamora, Esmeraldas and Machángara. Also, *E. coli* pathotypes were found in six rivers. Several physicochemical and metal parameters were detected in high levels, such as COD_TOTAL_ (in eight rivers), TSS (in six rivers), TS (in two rivers), Al (in nine rivers), Zn (in eight rivers), Pb (in three rivers), Cu (in three rivers), Fe (in two rivers), and Mn (in Machángara River). Our results agree with other studies in Latin America (such as Colombia, Brazil, and Peru) reporting similar contamination in water resources used for agriculture, livestock, and human consumption. Overall, Guayas, Guayllabamba, and Machángara Rivers showed the highest levels of physicochemical parameters (such as COD_TOTAL_ and TSS) and metal concentrations (such as copper, zinc, aluminum, iron, and manganese). Further studies should evaluate contamination sources and public health impact.

## Introduction

The ongoing discharge of untreated wastewater into the environment is a major concern worldwide. Even more so in developing countries, where untreated domestic wastewater is usually discharged into the nearest freshwater system, inducing severe impacts on ecosystems. Pollution in rivers leads to low yields of agricultural and industrial production^1^. Increased bacterial and chemical contamination contributes to severe problems in the food industry, since its production, processing, and distribution^2^. The continuous discharge of untreated effluents favors microbial proliferation (either commensal, opportunistic, or even pathogen microorganisms) and chemical contamination of surface water^3^, which is commonly used in rural areas as a drinking water source and for agriculture and livestock farming. This contamination eventually leads to serious public health risks and costs, such as the augmentation of chronic diseases and persistence of microorganisms with antibiotic resistance^4^, which is more evident in greater population density areas due to untreated domestic and industrial discharges^5^.

According to the United Nations Water Division, globally 80% of the domestic streams are discharged directly into rivers, lakes, and coastal zones without treatment, and Ecuador is not an exception^6^. This scenario represents a serious problem when surface water is used as an alternative to potable water, which currently occurs in numerous locations in Ecuador. Usually, in developed countries, potable water meets drinking water quality standards, being safe to drink or use for food preparation. However, in Ecuador, only 83% of the population has access to potable water, but may not always be drinkable quality water^7^. In rural regions, the situation is even worse, where only 53.9% of the population has potable water^7^. Numerous problems of access to drinking water lead part of the population to use river water for various domestic activities, including laundry, personal hygiene, and, on occasion, food preparation^8–10^. This national context led 28, 787 people to suffer, in 2015, from diarrhea and gastroenteritis due to a presumed infectious origin^11^. Some studies in Ecuador already postulated the contamination of water sources with potentially pathogenic microorganisms for human health^12–14^. These authors analyzed water resources through the general indicators of bacteriological quality, such as *Escherichia coli* and total coliforms^15^. Additionally, the contamination of surface waters by trace metallic elements due to mining supplies or industrial activities has been reported in several rivers located in the south of Ecuador, specifically in the localities of Nambija, Portovelo-Zaruma, and Ponce Enriquez. Due to the use of cyanide in mineral processing, water pollution was reported in several regions of Ecuador, through poor management of mining waste and conflicts related to regulations and policies^16,17^. So, the safety of these natural freshwater resources is also affected by various contaminants (trace metals, and major elements). These contaminants cause variations of the physicochemical properties of water resources, which directly influence microbial proliferation, and therefore physicochemical analysis is also an indispensable feature for the water quality assessment. Finally, high levels of heavy metals (such as Pb, Cr, Cu, and Zn) represent a serious public health risk because they are not biodegradable^18^.

Quito is the capital city of Ecuador with a population of 2,239,191 people based on the last census conducted in 2010^19^. Surprisingly, Quito has a small wastewater treatment plant (WWTP) in the southern part of the city, and, currently, 97% of domestic effluents are still being discharged directly into Machángara and Monjas Rivers without prior treatment^20^. In 2015, Voloshenko-Rossin et al. studied the water quality and the organic pollutants in the San Pedro–Guayllabamba–Esmeraldas watershed, while Benítez and colleagues characterized domestic wastewater samples from six different discharge points in the southern area of Quito^22^. In 2020, a study evaluated the quality of eighteen rivers located in Quito^23^, identifying Machángara and Monjas Rivers as the most contaminated rivers based on the physicochemical and microbiological parameters. However, little is still known about the microbial and chemical contamination in Ecuador’s main rivers, despite some studies recently realized in rivers of certain major cities (Guayaquil, Esmeraldas, and Quito) of Ecuador^21,22,24^.

Other potentially pathogenic microorganisms to human health and even food production should also be evaluated in the water quality assessment, such as *Pseudomonas*, *Shigella*, *Salmonella*, *Legionella*, and *Campylobacter* spp.^3^. Besides commensal *E. coli* quantification, as fecal contamination biomarker, the microbial load analysis should include the determination of certain *E. coli* pathotypes, more exactly, enteroaggregative *E. coli* (EAEC), enterohemorrhagic *E. coli* (EHEC), enteropathogenic *E. coli* (EPEC), and enteroinvasive *E. coli* (EIEC)^3^. In developing countries, *E. coli* pathotypes are responsible for numerous infections among the population, particularly, children under five years old^25^. Certain *E. coli* pathotypes are associated with the consumption of contaminated food and water. In Ecuador, the prevalence of EAEC, EHEC, EPEC, and EIEC are reported in single locations^26^, but few studies are characterizing their prevalence in space. So, by monitoring these *E. coli* pathotypes in natural freshwater resources, we aim to better understand *E. coli* transmission among Ecuadorian regions. These *E. coli* pathotypes contain both extended‐spectrum beta‐lactamase genes and virulence factors for intestinal and extraintestinal infections, which could eventually lead to a trade‐off between resistance and virulence of *E. coli* or other bacteria^27^. The dissemination of antibiotic resistance and virulence factors in natural environments is currently not well understood, and therefore needs to be clarified. These virulence factors can affect a wide range of cellular processes, such as cell–cell signaling, ion secretion, protein synthesis, mitosis, cytoskeletal structure, and mitochondrial function^27^. Presently, the microbial load evaluation in water samples uses classic and molecular methodologies. *E. coli* and total coliforms counting are typically obtained by classic techniques. Molecular techniques, such as polymerase chain reaction (PCR) or quantitative PCR (qPCR), could be an efficient complementary analysis, allowing a rapid detection and quantification of certain microorganisms in water samples^28^.

Our study aimed to analyze the physicochemical characteristics (including major and trace metallic elements) and microbiological quality of natural freshwater resources in twelve rivers located in urban areas of eleven provinces of Ecuador (Coastal, Andean, and Amazonian regions). All analyzed samples were collected from areas of high population densities located next to these rivers, allowing us to evaluate the current contamination panorama of the main rivers of Ecuador that could affect human health.

## Results

### *Escherichia coli* and total coliform counts

**Figure 1:**
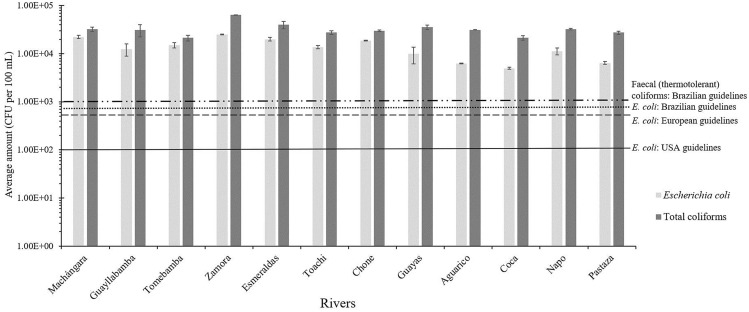
Average amount of *Escherichia coli* and total coliforms quantified in the rivers and their water classification accordingly to bathing-water standards by the USA, European and Brazilian guidelines. Legend: Threshold of faecal (thermotolerant) coliforms by Brazilian guidelines (**- -- -**); threshold of *E. coli* by Brazilian guidelines (**- - -**); threshold of *E. coli* by European guidelines (-- -- --); threshold of *E. coli* by USA guidelines (--------).

The counts of *Escherichia coli* and total coliforms were realized in the twelve rivers of the study set (Table 1). As shown in Fig. 1, all rivers showed concentrations of both *E. coli* and total coliforms above the maximum limits allowed by the United States of America (USA) standard values of the Recreational Water Quality Criteria^29^, European Union guidelines^30^, and Brazilian guidelines for bathing waters under Resolution CONAMA no. 274 of 29 November 2000^31^ (see Table S1 for additional information). Although microbial contamination was found in all rivers, the most polluted rivers were Zamora River in Loja at the southern Andean region (*E. coli*: 2.50 × 10^4^ CFU per 100 mL; and total coliforms: 6.38 × 10^4^ CFU per 100 mL), Esmeraldas River in Esmeraldas at the northeastern Coastal region of the country (*E. coli*: 2.00 × 10^4^ CFU per 100 mL; and total coliforms: 4.00 × 10^4^ CFU per 100 mL), and Machángara River in Quito at the central Andean region (*E. coli*: 2.25 × 10^4^ CFU per 100 mL; and total coliforms: 3.25 × 10^4^ CFU per 100 mL). Overall, the rivers of the Amazonian region showed the lower contamination levels of the present study, more exactly, Coca (*E. coli*: 5.00 × 10^3^ CFU per 100 mL; and total coliforms: 2.13 × 10^4^ CFU per 100 mL), Aguarico (*E. coli*: 6.25 × 10^3^ CFU per 100 mL; and total coliforms: 3.13 × 10^4^ CFU per 100 mL), and Pastaza (*E. coli*: 6.42 × 10^3^ CFU per 100 mL; and total coliforms: 2.75 × 10^4^ CFU per 100 mL) Rivers.

**Table 1:** Selection of the main Ecuadorian rivers and their collection samples for microbial, physicochemical, and metal analysis in this study.

Location	River	GPS Coordinates	City (Province)	Region	Collection sampling	Mean annual discharge (m^3^ s^-1^) ^a^	Monthly average temperature (°C)^b^	Annual Precipitation (mm) ^b^	Name of INAMHI Stations	GPS Coordinates of INAMHI Stations	Height of INAMHI Stations (m)
1	Machángara	0°14′03.6"S/78°30′53.0"W	Quito (Pichincha)	Andean	April 2016	4.2	N/A	1381.9	M0325 Garcia Moreno	0°14′5"S/78°37′38"W	1950
2	Guayllabamba	0°4′6,961″S/78° 22′21,87″W	Guayllabamba (Pichincha)	Andean	April 2016	N/A	N/A	847	M0345 Calderon	0°5′54″S/78°25′15″W	2645
3	Tomebamba	2°53′44.1″S/78°58′07.5″W	Santa Ana de los Cuatro Ríos de Cuenca, commonly referred as Cuenca (Azuay)	Andean	May 2016	21.37	N/A	878	M0426 Ricaurte-Cuenca	2°51′3″S/78°56′55″W	2545
4	Zamora	3°58′42.21″S/79°12′10.68″W	Loja (Loja)	Andean	June 2016	N/A	N/A	621.3	M0759 El Tambo-Loja	4°4′25″S/79°18′0″W	1580
5	Esmeraldas	0°56′31.3"N/79°38′34.5"W	Esmeraldas (Esmeraldas)	Coastal	July 2016	88.25	N/A	614.3	M0441 Sague (San Mateo)	0°53′13″S/79°37′54″W	15
6	Toachi	0°14′46.2″S/79°8′02,1″W	Santo Domingo (Santo Domingo de los Tsáchilas)	Coastal	July 2016	48.20	N/A	2792.4	M0348 Santa Anita-Km 10 Via Chone	0°13′50″S/79°14′54″W	560
7	Chone	0°41′41.6″ S/80°5′15.3″ W	Chone (Manabí)	Coastal	July 2016	N/A	25.32	1486.4	M0162 Chone—U. Catolica	0°39′51″S/80°2′11″W	13
8	Guayas	2°06′55.5"S/79°52′43.3"W	Guayaquil (Guayas)	Coastal	July 2016	1654.50	26.2	1064.5	M1096 Guayaquil—U. Estatal	2°12′0″S/79°53′0″W	6
9	Aguarico	0°03′36,8"N/76°52′25,0"W	Nueva Loja, also known as Lago Agrio (Sucumbios)	Amazonian	June 2016	N/A	23.8	4637.8	M1203 Lumbaqui	0°2′26″S/77°20′2″W	580
10	Coca	0°27′24,43″S/76°59′9,143″W	Puerto Francisco de Orellana, also known as El Coca (Orellana)	Amazonian	June 2016	32.23	25.5	3261.4	M1221 San Jose De Payamino	0°30′14″S/77°19′3″W	345
11	Napo	1°02′40.12″S/77°47′37.61″W	Tena (Napo)	Amazonian	June 2016	1105	N/A	2186.7	M0490 Sardinas	0°22′16″S/77°48′6″W	1615
12	Pastaza	1°27′05.8"S/78°09′18.6"W	Puyo (Pastaza)	Amazonian	June 2016	N/A	21.8	3557.1	M0041 Sangay (P. Santa Ana)	1°41′18″S/77°57′31″W	880

^a^ Data from the National Institute of Meteorology and Hydrology (INAMHI, http://www.serviciometeorologico.gob.ec/); ^b^ INAMHI. (2013). Anuario Metereológico. Retrieved from: https://www.serviciometeorologico.gob.ec/docum_institucion/anuarios/meteorologicos/Am_2013.pdf; N/A: Not available.

### Prevalence of bacterial genera and *Escherichia coli* pathotypes in river samples

Other growth media cultures were also assessed to detect several bacterial genera. In MacConkey agar, all water samples showed growth of Gram-negative rods, which can include *Escherichia*, *Salmonella*, *Shigella* (enteric bacteria), and *Pseudomonas* (non-enteric bacterium) genera. Yet, during the culture on Legionella CYE Agar Base, none of the rivers evidenced the growth of pure colonies, displaying bacterial contamination. No growth was detected on Salmonella-Shigella agar and Campylobacter agar in any water sample. Therefore, the presence or absence of these genera (*Legionella*, *Pseudomonas*, *Salmonella*, *Shigella*, and *Campylobacte*r spp.) was then evaluated through polymerase chain reaction (PCR) analysis. As suspected by growth media culture, none of the rivers revealed the presence of *Salmonella*, *Shigella*, or even *Campylobacter* spp., but all rivers showed the presence of *Pseudomonas* and *Legionella* spp. Finally, PCR analysis also evidenced the presence of EIEC pathotype in the Esmeraldas, Chone, Machángara, Guayllabamba, and Napo Rivers. EPEC pathotype was detected in the Zamora River and EAEC pathotype was also found in the Machángara River. However, the EHEC pathotype was not observed in any river.

### Analysis of physicochemical parameters

**Figure 2:**
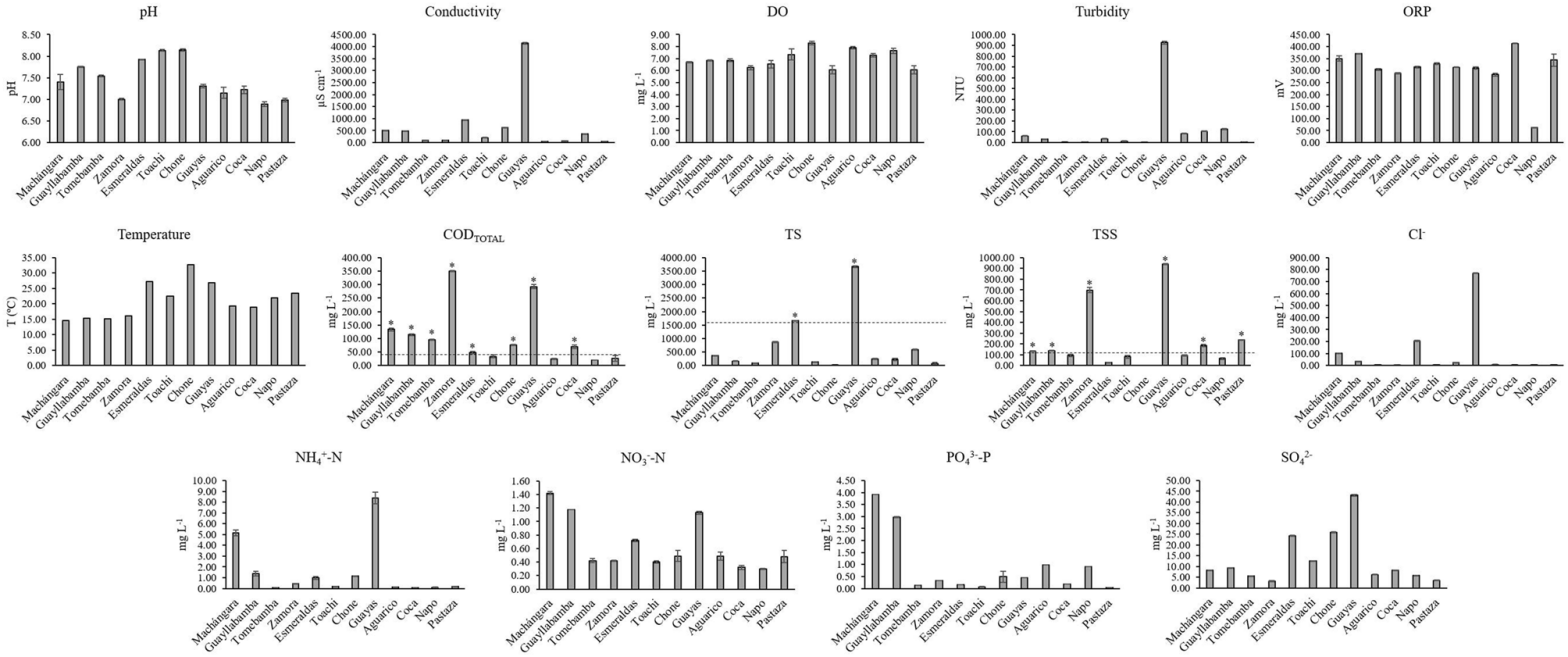
Average and standard deviation values of physicochemical parameters quantified in water samples of the twelve rivers in this study. Legend: Threshold of a certain physicochemical parameter (-—-); * exceedance values according to legislation.

Additionally, we analyzed the physicochemical parameters (Fig. 2). These parameters were compared to the maximum contaminant levels for the preservation of aquatic and wildlife in freshwater established in the Ecuadorian legislation^32^ (see Table S2 for additional information). The Chone River showed the highest temperature (32.7 ºC) while the Machángara River registered the lowest value (14.5 ºC). This is not surprising, as the Chone River is located in the Coastal region, where high ambient temperatures occur, and the Machángara River is located in the Andean region, at 2800 m.a.s.l. Values for pH (6.89–8.14), DO (6.08–8.30 mg L^−1^) and NO_3_^−^-N (0.30–1.42 mg L^−1^) were within the recommended ranges and illustrated the intrinsic natural variance due to the high geomorphological diversity between the three main regions of Ecuador (i.e., Coastal, Andean, and Amazonian)^32^.

High values of conductivity were found in Guayas (4137.33 µS cm^−1^), Esmeraldas (938.53 μS cm^−1^), and Machángara (501.10 µS cm^−1^) Rivers. The Environmental Protection Agency (EPA) suggests a range of conductivity between 150 and 500 μS cm^−1^, and therefore the conductivity values found in the river basins are higher than the recommended values^18^. In addition, the ORP values found in this study were between 62.44 and 412.77 mV, in which Coca River showed the highest value of ORP.

Ammonium levels ranged from 0.08 to 8.38 mg L^−1^, evidencing the highest value in the Guayas River (8.38 mg L^−1^), and followed by the Machángara River (5.15 mg L^−1^).

Regarding the total COD (COD_TOTAL_) values, it was found that eight rivers exceeded the value recommended by the Ecuadorian legislation (40 mg L^−1^)^32^. The Zamora River registered the highest COD_TOTAL_ value (349.73 mg L^−1^), followed by the Guayas (292.67 mg L^−1^) and the Machángara Rivers (133.58 mg L^−1^). The Guayllabamba, Tomebamba, Chone, Coca, and Esmeraldas Rivers exceeded the recommended COD_TOTAL_ value by a factor of 2.9, 2.4, 1.9, 1.7, and 1.2, respectively.

The Guayas and Esmeraldas Rivers from the Coastal region showed high concentrations of total solids (TS) with values of 3667.50 and 1657.50 mg L^−1^, respectively. These values are 2.3 and 1.1 times higher than the maximum allowable limit for discharges to water bodies established by Ecuadorian legislation^33^. Meanwhile, Guayas (939 mg L^−1^), Zamora (697.50 mg L^−1^), Coca (182.50 mg L^−1^), Pastaza (237.50 mg L^−1^), Machángara (132.50 mg L^−1^), and Guayllabamba (137.50 mg L^−1^) Rivers had TSS values above the maximum value (130 mg L^−1^) specified by Ecuadorian legislation for discharges to freshwater bodies^33^. Concentrations of sulphates (3.27–43.15 mg L^−1^), phosphates (0.04–3.91 mg L^−1^), and chlorides (0.07–769.58 mg L^−1^) were within the limits established by the Ecuadorian legislation^33^. However, in the case of chlorides, it is important to note that Machángara, Esmeraldas, and Guayas Rivers registered concentrations between one and two orders of magnitude higher than the remaining rivers.

**Figure 3:**
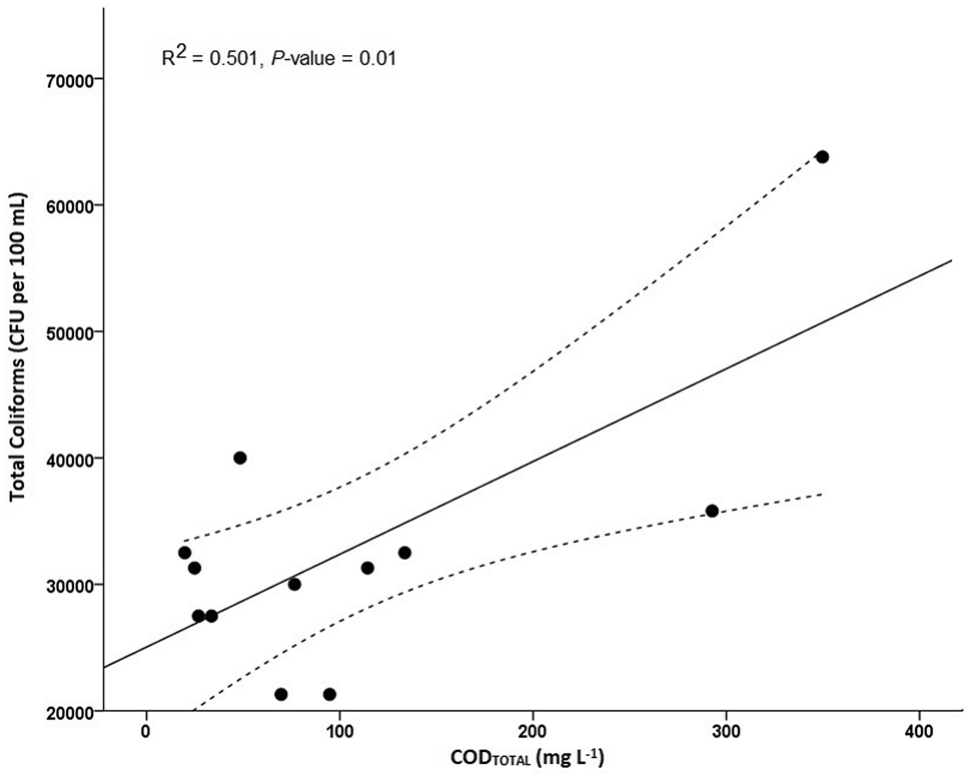
Linear logistic regression between total coliforms and COD_TOTAL_ (R^2^ = 0.501, *P*-value = 0.010; N = 12). Legend: Upper and lower 95% Confident Interval (95% CI) limit in the linear logistic regression (-—-).

Statistical analysis was performed between the concentration of *E. coli* and total coliforms against physicochemical parameters, using linear and multiple logistic regressions. Several correlation analyses were examined and we only found a statistically significant correlation between total coliforms and COD_TOTAL_ (R^2^ = 0.501, *P*-value = 0.010; N = 12). However, this correlation did not reveal a good fit (Fig. 3), which could be partially attributable to total coliforms as a variable. The Benjamini–Hochberg method was then used for multiple test corrections. The correlation between total coliforms and COD_TOTAL_ did not show statistical significance in the adjusted *P*-value (*P* = 0.089).

### Analysis of trace metals and major elements

**Figure 4:**
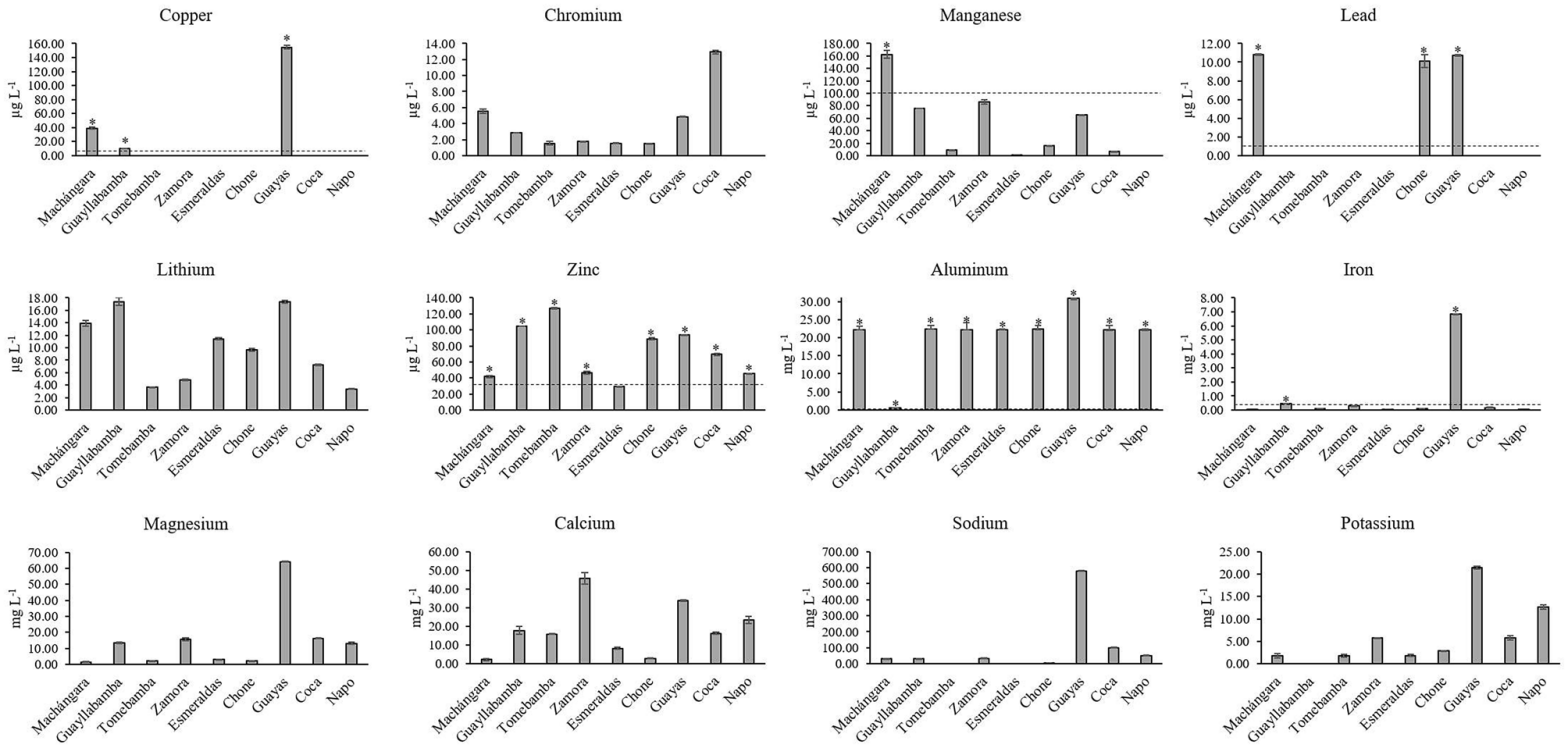
Average and standard deviation values of trace metals and major elements quantified in water samples of the nine rivers in this study. Legend: Threshold of a certain trace metal or major element (-—-); * exceedance values according to legislation.

It is worth mentioning that the samples from three rivers (Toachi, Pastaza, and Aguarico) were not analyzed for trace metals and major elements due to the cross-contamination of the samples during transport. Therefore, only nine rivers out of twelve were analyzed, as shown in Fig. 4. The following trace elements were analyzed: copper (Cu), chromium (Cr), manganese (Mn), lead (Pb), lithium (Li), and zinc (Zn); whereas the major elements were: aluminum (Al), iron (Fe), magnesium (Mg), calcium (Ca), sodium (Na), and potassium (K). Chromium (1.52–12.93 µg L^−1^) and Li (3.35–17.39 µg L^−1^) were the trace metals that were consistently below the limits (see Table S3 for additional information). Concentration ranges of Pb (10.12–10.82 µg L^−1^) and Cu (10.17–154.67 µg L^−1^) were the lowest measured in the surface water samples. The Chone, Machángara, and Guayas Rivers exceeded the maximum value of Pb by a factor of 10; while the Guayllabamba, Machángara, and Guayas Rivers exceeded the established value of Cu by a factor of 2.0, 7.8, and 30.9, respectively. The concentration of Zn (29.50–127.02 µg L^−1^) exceeded the limits established by Ecuador (30 µg L^−1^) in almost 100% of analyzed samples except for the Esmeraldas River. In the case of major elements, all rivers analyzed in this study evidenced Al levels exceeding the Ecuadorian threshold, in particular, the Guayas River (30.80 mg L^−1^), followed by Chone (22.45 mg L^−1^), Tomebamba (22.44 mg L^−1^), Esmeraldas (22.26 mg L^−1^), Zamora (22.25), and Machángara (22.17 mg L^−1^) Rivers. The concentrations of Fe were 1.5 and 22.8 times higher than the recommended values of Ecuadorian legislation (0.3 mg L^−1^) in Guayllabamba and Guayas Rivers, respectively. The highest concentrations of Fe and Al were observed in the same river (Guayas River). Mg (1.47–64.18 mg L^−1^), Ca (2.20–45.69 mg L^−1^), Na (4.85–578.82 mg L^−1^), and K (1.73–21.43 mg L^−1^) were also detected in high concentrations in several rivers. The Guayas River also registered the highest concentrations of Mg, Na, and K, while the highest concentration of Ca was detected in the Zamora River.

Finally, the statistical analysis did not find any correlation between metal concentrations and *E. coli* or total coliforms. Despite these results, we found a significant correlation between Fe and Cu concentrations (R^2^ = 0.986, *P*-value < 0.010; data not shown).

### Evaluation of the contamination between regions

**Figure 5:**
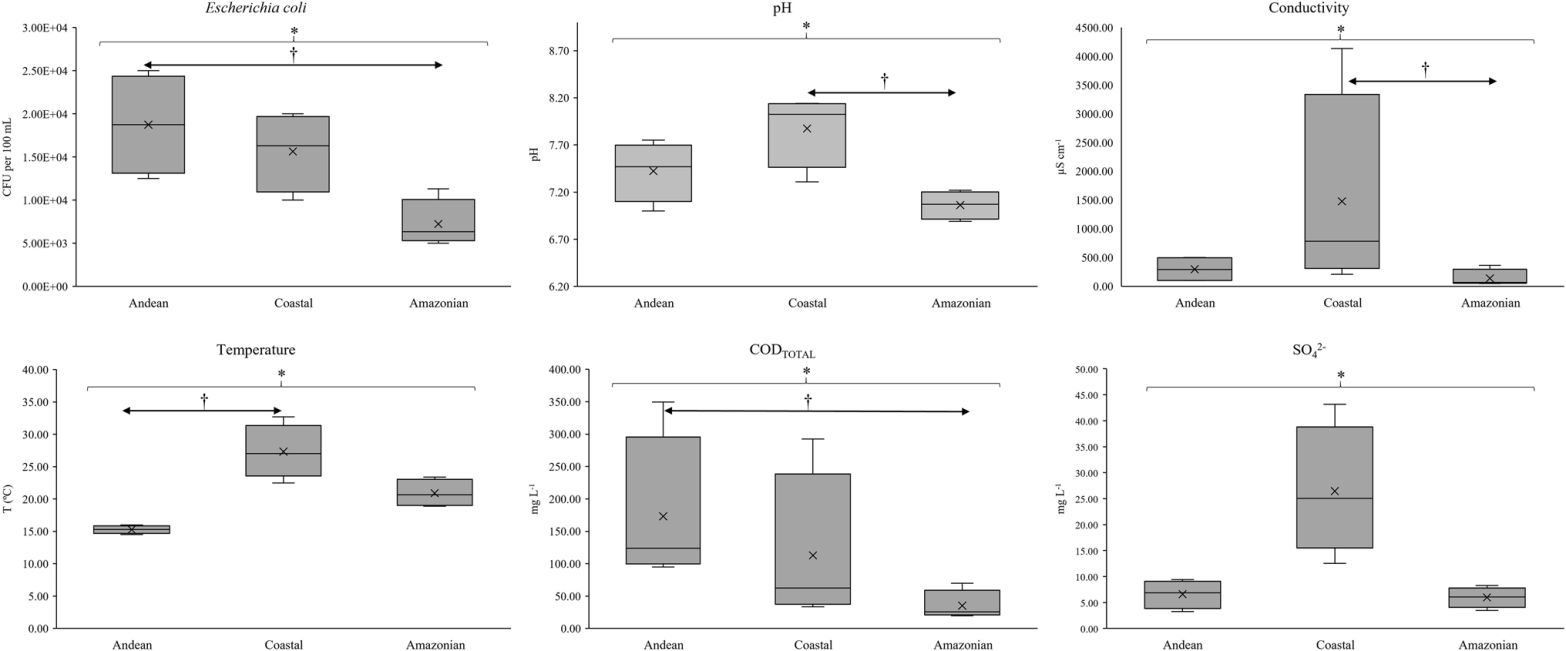
Statistical differences between regions (Andean, Coastal, and Amazonian) on microbial and physicochemical contamination in water samples of the present study. Legend: Statistical *P*-value obtained through Kruskal–Wallis non-parametric one-way analysis of variance (*P* < 0.050); † Statistical *P*-value obtained through Mann–Whitney test for paired comparisons (*P* < 0.050).

To better evaluate different river systems in the present study, all parameters (microbial, physicochemical, trace metals, and major elements) were further analyzed between different regions of Ecuador (Fig. S1 and Fig. S2 for additional information). So, the Kruskal–Wallis non-parametric one-way analysis of variance was used to compare contamination between regions (Andean, Coastal, and Amazonian), followed by a Mann–Whitney test for paired comparisons. Statistical differences were found on six parameters (*P*-values < 0.050; Fig. 5), more exactly, *E. coli* (*P* = 0.031), pH (*P* = 0.030), conductivity (*P* = 0.039), temperature (*P* = 0.010), COD_TOTAL_ (*P* = 0.030), and SO_4_^2−^ (*P* = 0.025). The Mann–Whitney paired comparisons demonstrated the following differences: *E. coli* concentrations between Andean and Amazonian regions (*P* = 0.032), showing average concentrations of 1.88 × 10^4^ and 7.24 × 10^3^ CFU per 100 mL, respectively; pH values between Coastal and Amazonian regions (*P* = 0.024), showing mean values of 7.88 and 7.06, respectively; conductivity values between Coastal and Amazonian regions (*P* = 0.032), showing mean values of 1476.46 and 137.14 μS cm^−1^, respectively; river temperatures between Coastal and Andean regions (*P* = 0.007), showing mean values of 27.33 and 15.28 ºC, respectively; and COD_TOTAL_ values between Andean and Amazonian regions (*P* = 0.024), showing mean values of 173.10 and 35.26 mg L^−1^, respectively. The Mann–Whitney paired comparisons did not evidence statistically differences in SO_4_^2−^ concentrations between regions. Although the average concentration of SO_4_^2−^ in the Coastal region (26.45 mg L^−1^) was higher than Andean and Amazonian regions (6.60 and 5.98 mg L^−1^, respectively), the adjusted *P* value was 0.056 against both regions. No statistically significant values were found among trace metals and major elements.

## Discussion

### Bacterial contamination in urban areas of the main Ecuadorian rivers

All rivers showed *E. coli* levels above standard concentrations for bathing-water recommended by the USA, European and Brazilian guidelines (Fig. 1), in concordance with other studies in Latin America, such as Colombia^34^, Mexico^4^, and Perú^35^. Most of the rivers in this study could be treated to produce drinking or bathing water, however, a drastic and expensive treatment would be necessary, being economically challenging in Ecuador.

Some studies in the USA reported lower levels of *E. coli* and total coliforms contamination than those reported in Latin America^36,37^. In particular, the study of Bower and colleagues^37^ demonstrated that 28 of the 74 analyzed samples did not exceed 235 CFU per 100 ml of *E. coli* showing a drastically lower level of contamination when compared to this study. In addition, other studies reported different levels of *E. coli* ranging from 3.1 × 10^5^ to 6.4 × 10^5^ CFU per 100 mL in Asia (India, Nepal and Iran), and 4.2 × 10^4^ to 5.4 × 10^4^ CFU per 100 mL in Spain^5,38,39^. Therefore, the contamination levels were higher than the results obtained in our study (5.00 × 10^3^ to 2.50 × 10^4^ CFU per 100 mL).

The selection of the sampling locations was an important step for the analysis of the water quality. In our study, all sampling locations were selected from dense urban areas and downstream of the most contaminated zones (Table 1). It is important to mention that the levels of total coliforms and *E. coli* were obtained at a similar order of magnitude, suggesting that most of the total coliforms were constituted by typical *E. coli* from animals and humans’ enteric origin*.* Most likely, these results evidenced environmental contamination of the rivers set by urban sewages, as previously reported^40^. Although all water samples were collected from areas of high population density, the contamination in our study was most probably due to the lack of wastewater treatment plants. Untreated sewage, combined with the geographical locations and the ambient temperatures, could contribute to the bacteria proliferation in surface waters^6^.

Next, we reported the presence of three *Escherichia coli* pathotypes (EAEC, EPEC, and EIEC). EHEC was not detected in any samples from our study. Although EHEC is one of the most prevalent *E. coli* pathotypes among environmental samples, Stanford et al. demonstrated seasonal variations in the prevalence of *E. coli* pathotypes^41^. The lack of positive EHEC results could be due to the cross-sectional study realized during a single season, showing one of the limitations of the present study. The EIEC was the most prevalent *E. coli* pathotype and it was found in five rivers. On the other hand, the EPEC and EAEC pathotypes were detected only once. More exactly, the EPEC was found in Zamora River while the EAEC was observed in Machángara River. These *E. coli* strains are more commonly found in rivers from developing countries, even in surface water resources^42^. *E. coli* pathotypes even on samples with low concentrations of total coliforms and *E. coli* constitute a greater threat to public health. All *E. coli* pathotypes are potentially dangerous to the population (particularly, in children). *E. coli* pathotypes may cause urinary tract infections, bacteremia, and bacterium-related diarrhea, being also the main cause of neonatal meningitis in humans and animals^25^. These findings represent a possible public health problem taking into account the type of distribution of the untreated water to the surrounding population, where the river water is usually used for numerous local practices (domestic, agricultural, live stocking, and even recreational activities). Currently, public health officials rely on infection reports by certain communities (such as indigenous, and rural communities) or public health outbreaks for assessing pathogen and/or chemical levels in water resources. So, future monitoring should be simultaneously realized in untreated wastewaters and natural freshwater resources. Finally, besides the standard quantification of *E. coli* and total coliforms, the detection of *E. coli* pathotypes could be useful as an additional indicator in water analysis to prevent waterborne disease outbreaks.

### Physicochemical parameters of surface waters

The majority of values found in the rivers were below the maximum limits established by the local legislation. However, certain parameters, such as TSS (132.5 to 939 mg L^−1^ > 130 mg L^−1^), COD_TOTAL_ (48.37 to 349.73 mg L^−1^ > 40 mg L^−1^), and TS (1657.50 to 3667.50 mg L^−1^ > 1600 mg L^−1^), were above Maximum Contaminant Levels (MCL; Fig. 2). In Ecuador, few studies assessed these chemical parameters in rivers ^21,24^. Voloshenko-Rossin and colleagues evaluated some physicochemical parameters in the San Pedro, Guayllabamba and Esmeraldas Rivers^21^, obtaining similar values of pH, conductivity, dissolved oxygen (DO), and turbidity when compared to our study. In Guayas, Damanik-Ambarita and colleagues studied the water quality of the Guayas River basin, evidencing also analogous values of pH, temperature, and DO. However, other physicochemical parameters were reported in lower levels when compared to our results, such as conductivity, turbidity, COD_TOTAL_, and TSS^24^. Other studies in Latin American countries also analyzed these basic parameters reporting similar levels of temperature, pH, and turbidity, such as Brazil^42^.

The high conductivity values were found in Guayas (4137.33 µS cm^−1^) and Esmeraldas (938.53 µS cm^−1^) Rivers. However, samples from the Guayas and Esmeraldas Rivers were collected in the urban area located near the Pacific Ocean, and so their high conductivity values could be associated with the presence of high concentrations of certain salts (such as Na and Mg) due to the entrance of sea waters. When measuring mixed water or saline water, conductivity values can easily achieve values greater than 5000 µS cm^−1^, in which case these rivers demonstrated normal conductivity values^32^. It is important to mention that samples from the Guayas River could have also shown a higher conductivity due to geological factors of the studied area, where it possesses clay soil. Therefore, it was expected to find high indices of conductivity among Guayas and Esmeraldas Rivers in opposite to rivers with granite associated soils (such as Toachi, Tomebamba, and Zamora Rivers), where this type of soil does not ionize and usually shows low conductivity values. In addition, brackish water samples with high conductivity values generally show higher values of TS and TSS, as previously detected in Guayas and Esmeraldas Rivers. Therefore, their higher TS and TSS values were considered normal among brackish systems^32^. On the other hand, DO values ​​were quantified between 6.08 and 8.30 mg L^−1^, being slightly above the minimum value allowed by the Ecuadorian Legislation (at least 6 mg L^−1^ or 80% saturation). It is important to mention that DO values could vary with temperature^12,18^, where higher temperatures usually diminished dissolved oxygen levels in the water. The dissolved O_2_ range measured in the rivers of this study was found to be suitable for natural waters depending on turbulence, temperature, salinity, and altitude^43^.

### Trace metals in surface waters

The majority of the elements were below the permitted limit for water aimed at agricultural use or for the preservation of aquatic and wildlife (Fig. 4)^32,44^. However, some levels of Cu, Pb, and Fe, and most levels of Zn and Al were the exceptions, showing high concentrations above the MCL at several sampling points. Although the maximum values recommended by the WHO are usually lower than the Ecuadorian legislation, it is important to mention that most of the elements were below both limits.

Nevertheless, Guayas and Machángara Rivers, indicators of the surface water quality of the two most populated cities of Ecuador (Guayaquil and Quito, respectively), and Chone River registered concentrations of Pb ten times higher than the maximum contaminant level (1 µg L^−1^). Lead is considered an important toxic heavy element in the environment, affecting almost every function in humans^45^. Even though lead is naturally present in the environment, anthropogenic activities (fossil fuels burning, mining, and manufacturing) contribute to its increase^45^. The Pb levels found in these three rivers were similar to the contamination levels reported by Cui et al. in urban zones of rivers in Northeast China (Harbin City)^46^. It is important to mention that the values of lead contamination in our study were very close to the limit of quantification (LOQ; 10.12 µg L^−1^). Therefore, it is plausible that these concentrations could not be accurately distinguished in these rivers. However, Machángara River already showed superior lead contamination (59.7 µg L^−1^) in a previous study^23^.

Cu was detected in the Guayas, Machángara, and Guayllabamba Rivers at concentrations exceeding the Ecuadorian guidelines. Similar contamination values of Cu were already reported in other countries, such as Bangladesh (50–100 µg L^−1^)^47^ and Canada (1–110 µg L^−1^)^48^. Some sources mentioned that these levels of Cu could be associated with the contamination from water pipes from households or industries^49^. However, other countries, such as Chile (170–630 µg L^−1^)^50^ and the USA (10–570 µg L^−1^)^51^, reported higher values of Cu on rivers. These higher concentrations could be explained by mining industries or activities near the water sources. Excess copper induces oxidative stress, DNA damage, and reduced cell proliferation leading to copperiedus^52^.

Although Zn is an essential element for all organisms, an excess of zinc plays a significant role in cytotoxic events in the cells. This element is involved in cell death of the brain, and its cytotoxicity induces ischemia or trauma^53^. In our study, eight rivers revealed Zn levels above the quality criteria^32^, ranging from 1.5 until 4.2 times higher than the MCL (30 µg L^−1^). These levels were still found below contamination levels from other studies realized in China^46^ and Brazil^54^. However, our levels of Zn are superior to the levels reported in Argentina^55^. These authors analyzed water samples from La Plata basin, showing levels of Zn between 0.2 and 11.9 µg L^−1^. Although their levels of Zn were below our results, these authors suggested that people would eventually experience high health risks through continuous consumption. So, these health risks are also plausible to the Ecuadorian population exposed to the rivers in our study.

Furthermore, Al and Fe were detected in values higher than those established by WHO (2011), by Ecuadorian legislation, or even in surface waters used for human consumption in the country^56^. As previously described, Al comes mainly from natural sources being one of the main constituents of the silicates that make up the mineral clay^57^. More exactly, Al concentrations were quantified between 0.49 and 30.80 mg L^−1^. Interestingly, seven rivers showed similar elevated Al concentrations (around 22 mg L^−1^). However, a previous study in Ecuador already reported analogous Al concentrations (17.30–18.25 mg L^−1^) in seven of eighteen rivers of Pichincha province^23^. These similar levels can probably be attributed to a strong build-up of Al from natural resources rather than directly from wastewater discharges due to anthropogenic activities. It is important to mention that Ecuador is a country famous for its large number of volcanoes contributing to the Al accumulation in soil and natural water resources^58^. Therefore, it is plausible that the high levels of Al in surface water on these locations did not differ significantly between them even with the anthropogenic activities in the urban areas of the rivers. Even though anthropogenic activities, such as discharge of industrial and domestic effluents, use of agricultural chemicals, land use, and cover changes, are typically the major factors that influence surface water quality. Accumulative exposure to this metal in low concentrations does not cause any harm to humans or animals. However, high concentrations of metals (such as Al) can trigger complications in the kidney due to metal accumulation and also induce cases of infertility in animals^58^ but its bioavailability depends on its species. Dissolved Al in water may induce risk for human health when reaching values for the internal aluminum load above 15 µg L^−1^ in urine or 5 µg L^−1^ in serum^59^. In Ecuador, the MCL of Al for the preservation of aquatic and wildlife in fresh and marine water is 0.1 mg L^−1^. Therefore, most rivers surpassed this legal value by more than 200 times, excepting the Guayllabamba River (approximately 5 times more than the MCL). The accumulative exposure of Al in these rivers could be potentially dangerous for aquatic life and even for human through regular water consumption. On the other hand, high concentrations of Fe were only detected in the Guayas (6.84 mg L^−1^) and Guayllabamba (0.46 mg L^−1^) Rivers, showing approximately a Fe concentration in the Guayas River of 10 times higher than the MCL (0.7 mg L^−1^) recommended by the World Health Organization^44^. Although this Fe concentration is not an immediate danger to public health, cumulative Fe contamination could cause hemorrhagic necrosis and disorders in the stomach mucosa^60^. So, further studies should monitor Fe variations in these rivers.

Previous studies^18,60^ reported similar metal analysis, showing also elevated concentrations of dissolved Fe, Mn, Al, Pb, and Zn. These large metal concentrations are usually associated with high soil erosion and discharges of contaminated water from different anthropogenic activities (such as industrial, oil, and agricultural), and followed by several public health issues in the surrounding communities, such as neurological problems, skin irritation, hormonal imbalances, atopic dermatitis, and thyroid problems^2^.

In Latin America, in the last decades, high concentrations of metals have been found in several rivers^2,42,60^. In Colombia, Cd and Pb were the highest metal values found nearby crops of vegetables and legumes^2^. These studies reported the contamination by several metals in water resources and warned for the use of these waters in the food industry (livestock and agriculture). Likewise, studies in the United States realized similar metal analysis in water supplies, showing significantly lower metal concentrations^51,61^. These low levels of metals in surface water could be due to the strict national regulations that control the heavy metal levels of effluents from large-scale industries^61^.

In summary, the main rivers of Ecuador showed unacceptable microbial, physicochemical, and metal levels for the preservation of aquatic and wildlife in freshwater, nor human consumption or bathing waters, and agriculture activities. To the best of our knowledge, this is the first study in Ecuador that simultaneously analyzed the microbial and physicochemical parameters in the three main natural regions (Coastal, Andean, and Amazonian), demonstrating statistically significant differences between these regions. However, this statistical analysis should be validated in future studies with a greater number of samples. Also, it is important to mention that there are some major limitations of the present study: (1) it is a cross-sectional study, and therefore unable to evaluate seasonal variations of microbial and physicochemical levels, (2) all physicochemical analyses were realized using water samples taken once in each river, (3) the sampling points were selected close to the main cities or even in urban areas, and it would be useful to extend this monitoring downstream in order to evaluate the extension of the observed contamination, and (4) this study only evaluated the presence or absence of several bacterial genera through PCR analysis without sequencing analysis.

Despite the increasing legislation in Ecuador, there is still an exceedance of the established standards, which suggests that practical control on effluent levels is underdeveloped. Finally, it is essential to evaluate a future scenario of reversing these high rates of microbial and chemical contamination by installing efficient wastewater treatment plants.

## Material and methods

### Sample site and collection

**Figure 6:**
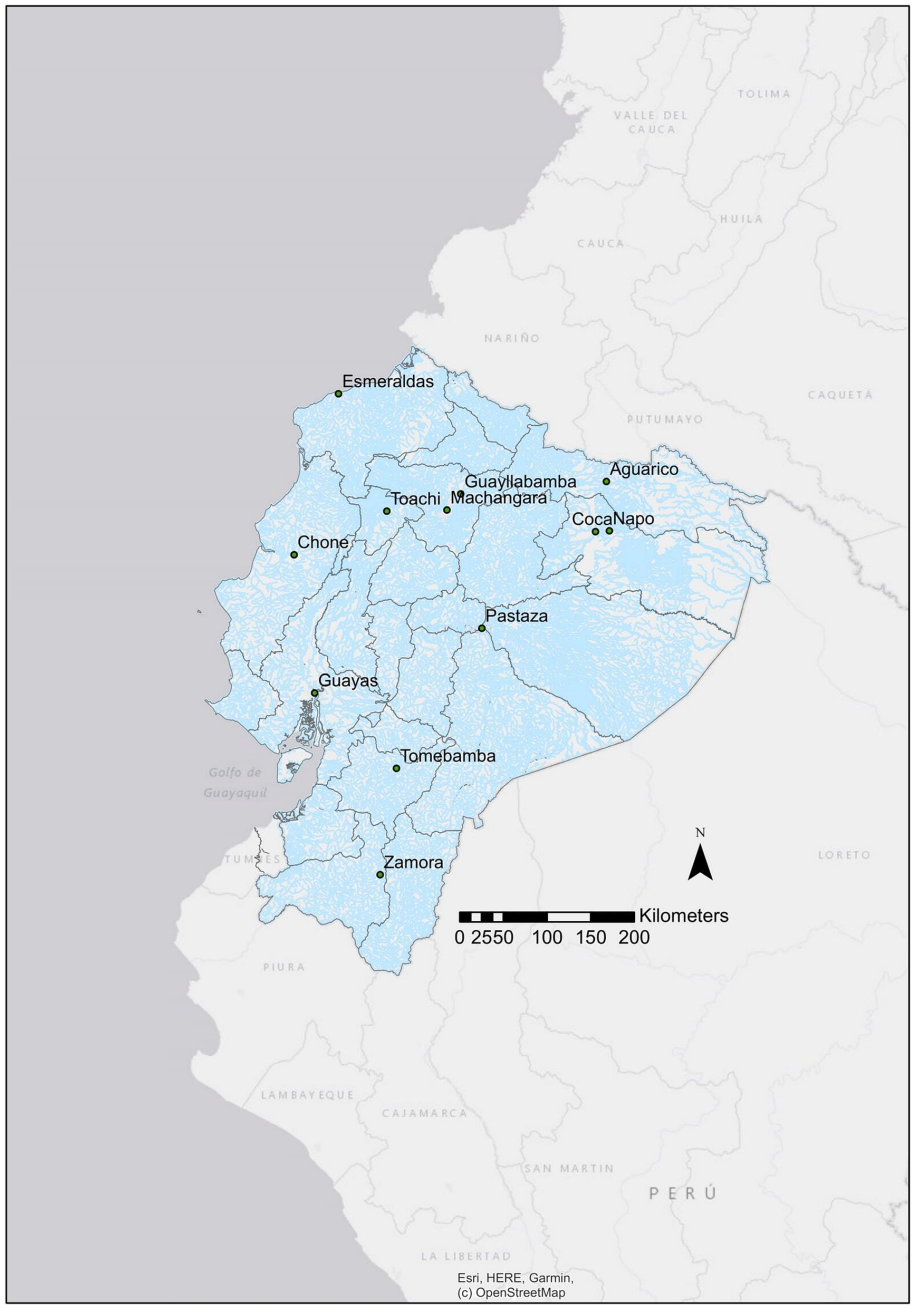
Illustration of the collection sampling points selected in this study for the microbial and chemical evaluation of the main Ecuadorian rivers. Legend: The map of Ecuador with the collection sampling points was created through ArcGIS Desktop software (version 10.8, available online: https://desktop.arcgis.com/es/).

Surface water samples were taken from rivers located along with eleven provinces of Ecuador (Fig. 6), twelve rivers were selected due to their proximity to high-populated cities and their location in the three geomorphologic regions (Coastal, Andean, and Amazonian). All samples were collected from urban sites, where the population lived close to the rivers. Water samples for the microbial analysis were taken on three different dates of collection during a month in each river (Table 1), while water samples for the physicochemical and metal analysis were collected only once on the last collection date. All microbial and analytical methods described below are similar to our previous publication realized by Borja et al.^23^ and reference publications cited in each subsection. For microbial analysis, samples were taken in previously sterilized glass containers by autoclaving at 121 ºC for 15 min. A total volume of 800 mL was collected from each river. Additionally, for the physicochemical analysis, water samples were taken once in each river, between April and July 2016, during the high-water stage for Amazonian Rivers, and the values of each parameter were obtained by triplicate measurements of each analyzed river sample. For chemical analysis and trace metallic elements, surface water samples were collected in amber glass bottles cleaned in a muffle oven at 550 ºC and in acid clean 1 L Teflon bottles previously washed with 10% hydrochloric acid and later rinsed with distilled water, respectively. Dissolved and suspended phases were separated immediately after collection by vacuum filtration using a 0.45 µm cellulose pre-cleaned filter. For metal analysis, the filtrate was transferred to acid cleaned high-density polyethylene Nalgene bottles and preserved with high purity concentrated nitric acid (HNO_3_; Merck, Massachusetts, USA) to obtain a final concentration of 2% v/v at the Laboratory of Environmental Engineering at Universidad San Francisco de Quito USFQ (LIA-USFQ).

### Sample preparation

Surface water samples (800 mL) were filtered through a nitrocellulose membrane 0.45 μm (Millipore) into a vacuum pump under aseptic conditions (Chemical Duty Pump, Millipore Inc.). Then, the following procedure was adapted from a previous study realized by Dobrowsky and colleagues with slight modifications^3^. Briefly, the membrane was removed and placed in a sterile falcon tube with 20 mL of distilled water. The tube was vortexed over 15 min to suspend the particles and microorganisms. The membrane was removed, and the tubes were centrifuged at 5000 rpm for 15 min to precipitate the sediments. The obtained pellet was suspended in 500 μL of sterile distilled water. Subsequently, this sample was then divided for bacterial DNA extraction through Power Soil Extraction Kit (Mo Bio Laboratories Inc.) and for bacterial growth cultures.

### Cultivation, quantification, and isolation of dominant bacteria from river samples

Different media cultures were employed to isolate or count the most diverse microorganisms in the samples. More precisely, a volume of 50 μL of the previous aliquot (pellet sample suspended in sterile distilled water) was incubated on MacConkey agar (Difco Laboratories Inc.) at 37 ºC for 18 to 24 h for the recovery of the genus *Escherichia*; on Salmonella-Shigella agar (Difco Laboratories Inc.) for the cultivation of *Salmonella* and *Shigella* genera at same conditions; on Legionella CYE agar (Difco Laboratories Inc.) at 35 ºC for 48 h to isolate *Legionella* spp.; and on Campylobacter agar for the isolation of *Campylobacter* spp. at 37 ºC for 18 to 24 h. Finally, for the quantification of *Escherichia coli* and total coliforms, successive dilutions of the initial aliquot were cultured in Chromocult agar medium (Biolab Laboratories, Merck Inc.) through classic dilution method^62^, and the results were obtained after 24–48 h of culture.

### DNA extraction

DNA from the microbial community in water samples was extracted following the instructions of the commercial PowerSoil DNA Isolation Kit (Mo Bio Laboratories Inc.). Briefly, 250 µL of the pellet obtained from the river water filtration was placed in the PowerBead tubes. The PowerBead tubes contained a buffer that allowed to disperse the soil particles and to dissolve humic acids while protecting nucleic acids from degradation. Later, solution C1 was placed, which contained sodium dodecyl sulfate (SDS) and other disruption agents required for complete cell lysis. Then, a step of 20 min vortexing was performed for complete homogenization and cell lysis in the samples. Subsequently, the tubes were centrifuged at 10,000 × *g* for 30 s at room temperature. A total volume of 500 μL of the supernatant was taken and placed in a 2 mL Collection Tube, afterward 250 μL of solution C2 was added, and the total volume in the tubes was incubated at 4 ºC for 5 min. Solution C2 contained a flocculant mixture (a combination of ammonium acetate, magnesium chloride (MgCl), ferric chloride (Fe(Cl), a salt of iron, a salt of aluminum, calcium chloride (CaCl), polyacrylamide, aluminum ammonium sulphate, and derivates) to precipitate non-DNA organic and inorganic material including humic substances, cell debris, and proteins. The tubes were centrifuged at 10,000 × *g* for 30 s at room temperature. 600 mL of supernatant from each tube was transferred to a new 2 mL Collection tube with 200 μL of solution C3. Solution C3 allowed to precipitate additional non-DNA organic and inorganic material. The tubes were centrifuged at 10,000 × *g* for 30 s at room temperature and 750 μL of the supernatant was mixed with 1.2 mL of Solution C4 (a high concentration salt solution). Half volume was placed inside Spin Filter and centrifuged at 10,000 × *g* for one minute at room temperature. Afterward, the liquid was discarded and the previous step was repeated twice with the remaining volume. In the next step, 500 μL of the C5 solution was added inside the Spin Filter, centrifuged at 10,000 × *g* for 30 s at room temperature, and discarded the liquid in each tube. The tubes were again centrifuged at 10,000 × *g* for 1 min at room temperature, removing the residual solution C5. Carefully the Spin Filter was placed on a new 2 mL Collection Tube. Finally, 100 μL of solution C6 sterile elution buffer was added to the center of the filter membrane. Then the tubes were centrifuged for 30 s at 10,000 × *g* and the Spin Filter was discarded. The DNA solution of each tube was stored at − 20 °C for further PCR analysis.

### Molecular identification of bacterial genera

Once the genomic DNA had been extracted from the different samples, specific primer pairs from previous studies were employed to identify several bacterial genera by polymerase chain reaction (PCR; see Table S4 for additional information). The PCR mixtures consisted of a final volume of 20 μL, containing 4 μL of 5X Green GoTaq Flexi buffer (1X final concentration; Promega, Madison, USA), 1.6 μL of MgCl_2_ (2.0 mM final concentration; Promega, Madison, USA), 0.2 μL of dNTP Mix (0.1 mM final concentration; Promega, Madison, USA), 1.0 μL of each PCR primer (0.5 μM final concentration; Table S4), 0.3 μL of GoTaq Flexi DNA polymerase (1.5 U final concentration; Promega, Madison, USA), 2 μL of template DNA, and the remaining volume of DNA-free water. For *Shigella* and *Salmonella* spp., the same PCR mix was used, with the exception that 0.2 μL of each primer (0.1 μM) was added. For *Pseudomonas*, *Legionella*, and *Campylobacter* spp., the same reaction mixture was used, with the exception that 0.8 μL, 1.0 μL, and 0.6 μL of each primer (0.3 μM) was added, respectively. The PCR analysis was performed in a thermocycler (Bio-Rad Laboratories Inc.) with the standard procedure illustrated in Table S4. The respective use of negative (without DNA sample and samples with other DNA-related bacteria) and positive (collection of identified strains of each genus or species through DNA sequencing) controls were used in each PCR assay. These positive controls were provided by the Microbiology Institute at Universidad San Francisco de Quito (MI-USFQ). All samples were randomly performed in triplicate with different negative and positive controls.

### Molecular identification of *Escherichia coli* pathotypes

For the molecular identification of *E. coli* pathotypes, the PCR mixtures consisted of a final volume of 20 μL, containing 4 μL of 5X Green GoTaq Flexi buffer (1X final concentration; Promega, Madison, USA), 2 μL of MgCl_2_ (2.5 mM final concentration; Promega, Madison, USA), 0.4 μL of dNTP Mix (0.2 mM final concentration; Promega, Madison, USA), 0.5 μL of GoTaq Flexi DNA polymerase (2.5 U final concentration; Promega, Madison, USA), 2 μL of template DNA, and the remaining volume of each PCR primer and DNA-free water. Volumes of 0.6 μL for EAEC, 1 μL for EHEC, 0.5 μL for EPEC, and 0.8 μL for EIEC were added of each PCR primer set (0.5 μM final concentration; see Table S5 for additional information). The positive control pathotypes (previously sequenced *E. coli* isolates, such as EHEC O157:H7 and EAEC 3591–87) were provided by the MI-USFQ from the microbial collection. All samples were randomly performed in triplicate with different negative and positive controls.

### PCR product analysis

The PCR products were visualized using electrophoresis in 2% agarose gels and staining with ethidium bromide 0.1%, with the respective use of negative and positive controls.

### Analytical methods

Physicochemical characterization of water samples was conducted, as described previously by Benitez et al. (2018) and Grube et al. (2020) according to US Standard Methods from the American Public Health Association^14,22^. Dissolved oxygen (DO), temperature (SM 4500-O A), conductivity (SM 2510), and pH (SM 4500 H^+^) were measured in situ with a portable multiparameter and corresponding probes (Thermo Fisher Scientific Model A329, Waltham, USA). Turbidity (EPA 180.1 Rev 2.0) was measured using a portable turbidimeter (Thermo Fisher Scientific AQUAFast AQ4500, Waltham, USA). Ammonium (NH_4_^+^; SM 4500-NH_3_), nitrate (NO_3_^−^; SM 4500-NO_3_^−^D), and chlorides (Cl^−^; SM 4500 Cl^−^D) were measured using an ion-selective electrode (Thermo Specific Ion Selective Electrode, ISE Orion). A calibration curve between potential (mV) and concentrations (R^2^ = 0.99) was constructed for every test. Chemical oxygen demand (COD_TOTAL_; SM 5520) and phosphates (PO_4_^3−^; SM 4500-P B) were measured by a colorimetric method, using a Spectronic 20D + spectrophotometer (Thermo Fisher Scientific, Waltham, USA). Sulphates (SO_4_^2−^; SM 426 C) were measured following filtrations, using Whatman glass microfiber filters (Grade 934-AH). Total solids (TS) (SM 2540 B) and total suspended solids (TSS) were measured using 0.45 µm cellulose filters, and dried in a 40 GC Lab Oven. Metal analysis on filtered and acidified water samples was conducted with a ThermoScientific iCAP 7400 ICP-OES at the LIA–USFQ. Standard solutions were prepared in dilute nitric acid from commercial standards (Sigma Aldrich, Trace–CERT multielement standard solution 6, USA). The detection and quantification limits were calculated by analyzing blank samples with at least 8 replicates, adding the average of the blank values with 3 and tenfold the standard deviation to obtain the limit of detection (LOD) and the limit of quantification (LOQ), respectively.

### Quality assurance/quality control

Quality control in metals analysis was conducted employing CRM 1640a–Trace elements in natural waters (National Institute of Standards and Technology, Gaithersburg, USA), which was measured every 10 samples (see Table S6 for additional information). Recovery percentages were calculated to determine matrix effects and measurements accurateness, and all concentrations were corrected based on the percentage recoveries. The recoveries varied between 89.43 and 105.42% for nickel and calcium, respectively.

### Statistical analysis

The data obtained from the microbial and physicochemical analysis of the water samples was analyzed by using the statistical software SPSS version 23.0 package. Linear and multiple logistic regressions were performed between the concentration of *E. coli* and coliforms, physicochemical parameters, and metal concentrations. To evaluate the relevance of the correlations, *P*-values were then adjusted for multiple testing using the Benjamini–Hochberg (BH) method^63^ implemented in RStudio software (version 1.3.1073; https://rstudio.com/). Adjusted *P*-values by the BH method were obtained using the option method =  ~ BH ~ of the p.adjust function from the stats base R package (Package stats version 4.1.0). In all hypothesis tests, a significant level of 5% was used as the standard. Also, differences in contamination between Andean, Coastal, and Amazonian regions were evaluated using Kruskal–Wallis non-parametric one-way analysis of variance with Mann–Whitney test for paired comparisons. In all tests, a *P*-value below 0.05 was considered a statistically significant value.

### Ethical approval and consent to participate

The study did not require approval from the research ethics committee as it did not involve human subjects or records.

### Consent to publish

The authors declare that they consented to publish the present study.

## Supplementary Information

Supplementary Information 1.

Supplementary Information 2.

Supplementary Information 3.

Supplementary Information 4.

Supplementary Information 5.

Supplementary Information 6.

Supplementary Information 7.

Supplementary Information 8.

## Data Availability

The data that support the findings of this study are available on request from the corresponding author and Supplementary Information.
