# Supplementary figures and images for: Determining the microbial and chemical contamination in Ecuador’s main rivers
Source: Sci Rep. 2021 Sep 3;11:17640. doi: 10.1038/s41598-021-96926-z (PMC8531378; doi:10.1038/s41598-021-96926-z)

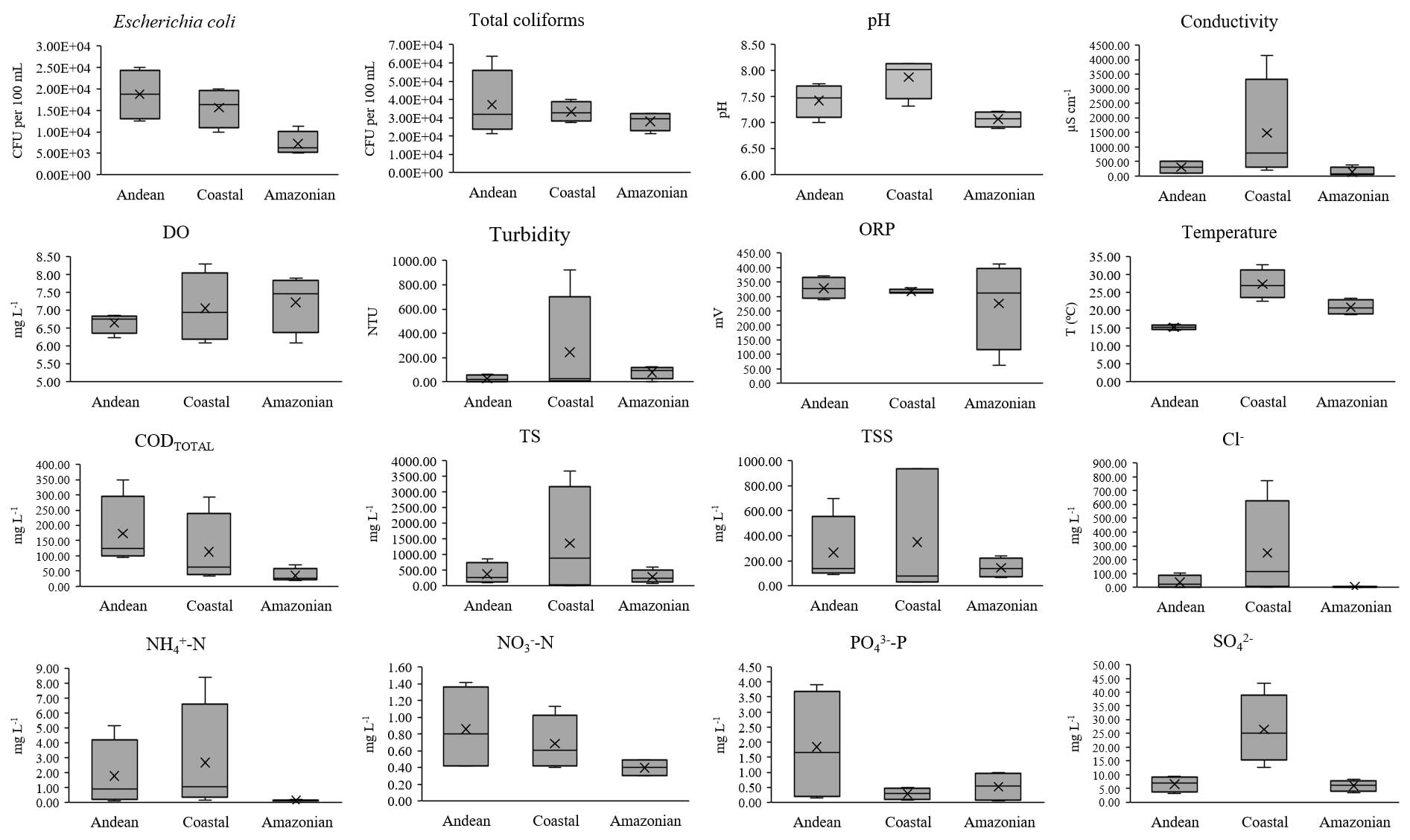

Supplement: Supplementary file 1 — Supplementary Information 1. [file 41598_2021_96926_MOESM1_ESM.jpg]

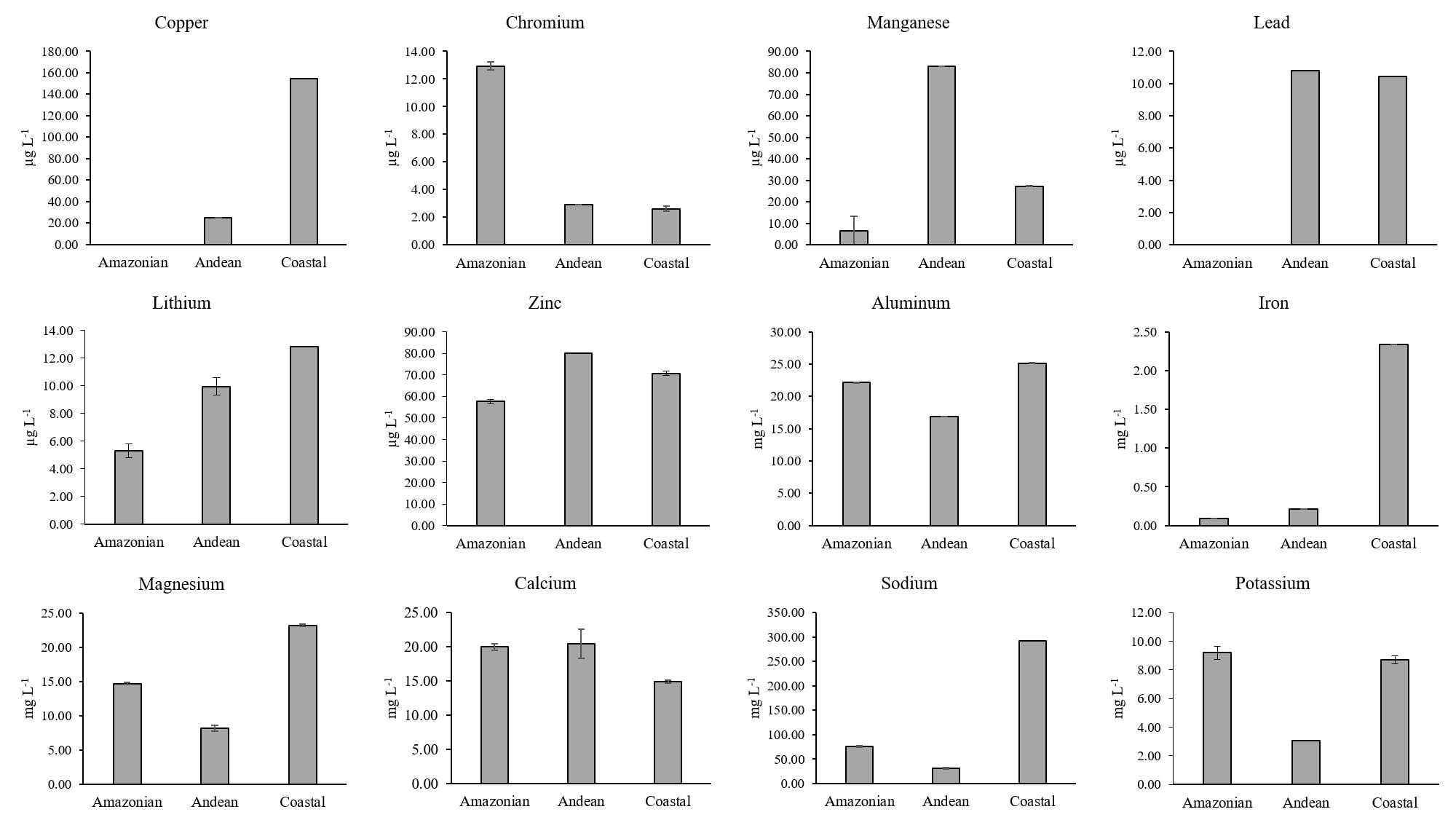

Supplement: Supplementary file 2 — Supplementary Information 2. [file 41598_2021_96926_MOESM2_ESM.jpg]
