# Supplementary material for: Determining the microbial and chemical contamination in Ecuador’s main rivers
Source: Sci Rep. 2021 Sep 3;11:17640. doi: 10.1038/s41598-021-96926-z (PMC8531378; doi:10.1038/s41598-021-96926-z)
Supplement: Supplementary file 3 — Supplementary Information 3. [file 41598_2021_96926_MOESM3_ESM.docx]

Manuscript title: **Determining the microbial and chemical contamination in Ecuador’s main rivers**

Authors: Dayana Vinueza, Valeria Ochoa- Herrera, Laurence Maurice, Esteban Tamayo, Lorena Mejía, Eduardo Tejera, and António Machado

**Supplementary Information**

**Table S1 – Average amount of *Escherichia coli* and total coliforms in the rivers and water classification** **applied to bathing-water standards by the USA, European and Brazilian guidelines.**

| **River**  **(GPS Coordinates)** | ***Escherichia coli***  **(CFU per 100 mL ± SD)** | **Total coliforms  (CFU per 100 mL ± SD)** | **USA guidelines**  **(*E. coli*: ≤100-126 CFU per 100 mL^a^;**  **No values are given for total coliforms)** | **European guidelines**  **(*E. coli*: ≤500 CFU per 100 mL^b^;**  **No values are given for total coliforms)** | **Brazilian guidelines**  **(*E. coli*: ≤800 CFU per 100 mL; Faecal (thermotolerant) coliforms: ≤1000 CFU per 100 mL^c^; No values are given for total coliforms:)** |
| --- | --- | --- | --- | --- | --- |
| Esmeraldas  (0°57'42.0"N / 79°37'51.7"W) | 2.00 × 10^4^ ± 1.77 × 10^3^ | 4.00 × 10^4^ ± 7.07 × 10^3^ | Not acceptable | Not acceptable | Not acceptable |
| Toachi  (0°14'46.2''S / 79°8'02,1''W) | 1.38 × 10^4^ ± 1.12 × 10^3^ | 2.75 × 10^4^ ± 2.26 × 10^3^ | Not acceptable | Not acceptable | Not acceptable |
| Chone  (0°41'41.6'' S / 80°5'15.3'' W) | 1.88 × 10^4^ ± 3.64 × 10^2^ | 3.00 × 10^4^ ± 1.09 × 10^3^ | Not acceptable | Not acceptable | Not acceptable |
| Guayas  (2°06'55.5"S 79°52'43.3"W) | 1.00 × 10^4^ ± 3.82 × 10^3^ | 3.58 × 10^4^ ± 3.72 × 10^3^ | Not acceptable | Not acceptable | Not acceptable |
| Machángara  (0°14'03.6"S / 78°30'53.0"W) | 2.25 × 10^4^ ± 1.77 × 10^3^ | 3.25 × 10^4^ ± 3.54 × 10^3^ | Not acceptable | Not acceptable | Not acceptable |
| Guayllabamba  (0°4'6,961''S/78° 22'21,87''W) | 1.25 × 10^4^ ± 3.54 × 10^3^ | 3.13 × 10^4^ ± 8.84 × 10^3^ | Not acceptable | Not acceptable | Not acceptable |
| Tomebamba  (0°27'24,43''S/76°59'9,41''W) | 1.50 × 10^4^ ± 1.94 × 10^3^ | 2.13 × 10^4^ ± 3.07 × 10^3^ | Not acceptable | Not acceptable | Not acceptable |
| Zamora  (0°27'24,43''S/76°59'9,125''W) | 2.50 × 10^4^ ± 4.43 × 10^2^ | 6.38 × 10^4^ ± 5.92 × 10^2^ | Not acceptable | Not acceptable | Not acceptable |
| Aguarico  (0°03'36,8"N / 76°52'25,0"W) | 6.25 × 10^3^ ± 1.41 × 10^2^ | 3.13 × 10^4^ ± 3.32 × 10^2^ | Not acceptable | Not acceptable | Not acceptable |
| Coca  (0°27'24,43''S/76°59'9,143''W) | 5.00 × 10^3^ ± 2.50 × 10^2^ | 2.13 × 10^4^ ± 2.25 × 10^3^ | Not acceptable | Not acceptable | Not acceptable |
| Napo  (0°27'24,43''S/76°59'9,21''W) | 1.13 × 10^4^ ± 1.77 × 10^3^ | 3.25 × 10^4^ ± 1.06 × 10^3^ | Not acceptable | Not acceptable | Not acceptable |
| Pastaza  (1°27'05.8"S/ 78°09'18.6"W) | 6.42 × 10^3^ ± 4.23 × 10^2^ | 2.75 × 10^4^ ± 2.16 × 10^3^ | Not acceptable | Not acceptable | Not acceptable |

Legend: SD – Standard deviation values; ^a^ Recreational Water Quality Criteria U.S. EPA, 1976. ^b^ Council of the European Union (2006). "Directive 2006/7/EC of the European Parliament and of the Council of 15 February 2006 concerning the management of bathing water quality and repealing Directive 76/160/EEC"). ^c^ Brazilian guidelines for bathing waters established by Resolution CONAMA n° 274 of 29 November 2000.
