# Supplementary material for: Determining the microbial and chemical contamination in Ecuador’s main rivers
Source: Sci Rep. 2021 Sep 3;11:17640. doi: 10.1038/s41598-021-96926-z (PMC8531378; doi:10.1038/s41598-021-96926-z)
Supplement: Supplementary file 4 — Supplementary Information 4. [file 41598_2021_96926_MOESM4_ESM.docx]

Manuscript title: **Determining the microbial and chemical contamination in Ecuador’s main rivers**

Authors: Dayana Vinueza, Valeria Ochoa- Herrera, Laurence Maurice, Esteban Tamayo, Lorena Mejía, Eduardo Tejera, and António Machado

**Supplementary Information**

**Table S2** **–** **Average and standard deviation** **values of physicochemical parameters of the twelve rivers analyzed in this study.**

| **River** | **pH** | **Conductivity** | **DO** | **Turbidity** | **ORP** | **T (ºC)** | **COD_TOTAL_** | **TS** | **TSS** | **Cl^-^** | **NH_4_^+^-N** | **NO_3_^-^-N** | **PO_4_^3-^-P** | **SO_4_^2-^** |
| --- | --- | --- | --- | --- | --- | --- | --- | --- | --- | --- | --- | --- | --- | --- |
|  |  | **(µS cm^-1^)** | **(mg L^-1^)** | **(NTU)** | **(mV)** |  | **(mg L^-1^)** | **(mg L^-1^)** | **(mg L^-1^)** | **(mg L^-1^)** | **(mg L^-1^)** | **(mg L^-1^)** | **(mg L^-1^)** | **(mg L^-1^)** |
| **MCL** | 6.5 - 9^a^ | N/A | N/A | N/A | N/A | N/A | 40^a^ | 1600^b^ | 130^b^ | 1000^b^ | N/A | 13^a^ | 10^b^ | 1000^b^ |
| **Esmeraldas** | 7.92 ± 0.01 | 938.53 ± 13.55 | 6.53 ± 0.30 | 34.60 ± 3.73 | 314.97 ± 3.71 | 27.30 | 48.37 ± 3.80* | 1657.50 ± 17.68* | 27.50 ± 3.54 | 204.91 ± 5.85 | 0.98 ± 0.15 | 0.72 ± 0.02 | 0.15 ± 0.00 | 24.24 ± 0.18 |
| **Toachi** | 8.13 ± 0.02 | 206.47 ± 1.43 | 7.34 ± 0.45 | 13.47 ± 1.04 | 328.53 ± 3.23 | 22.50 | 33.61 ± 5.69 | 127.50 ± 17.68 | 80.00 ± 14.14 | 0.07 ± 0.09 | 0.17 ± 0.01 | 0.40 ± 0.02 | 0.07 ± 0.01 | 12.56 ± 0.01 |
| **Chone** | 8.14 ± 0.03 | 623.50 ± 0.26 | 8.30 ± 0.16 | 5.30 ± 0.67 | 313.53 ± 1.59 | 32.70 | 76.56 ± 1.90* | 5.00 ± 0.00 | N/A | 24.23 ± 1.91 | 1.16 ± 0.02 | 0.49 ± 0.08 | 0.49 ± 0.23 | 25.84 ± 0.27 |
| **Guayas** | 7.31 ± 0.04 | 4137.33 ± 41.53 | 6.08 ± 0.34 | 925.00 ± 13.11 | 310.93 ± 4.82 | 26.80 | 292.67 ± 7.59* | 3667.50 ± 31.82* | 939 ± 7.07* | 769.58 ± 3.55 | 8.38 ± 0.53 | 1.13 ± 0.02 | 0.46 ± 0.00 | 43.15 ± 0.35 |
| **Machángara** | 7.40 ± 0.17 | 501.10 ± 1.56 | 6.69 ± 0.03 | 60.50 ± 2.46 | 349.90 ± 10.61 | 14.50 | 133.58 ± 4.80* | 370.00 ± 4.71 | 132.50 ± 3.54* | 104.12 ± 1.63 | 5.15 ± 0.26 | 1.42 ± 0.02 | 3.91 ± 0.00 | 8.23 ± 0.01 |
| **Guayllabamba** | 7.75 ± 0.01 | 474.63 ± 1.22 | 6.84 ± 0.05 | 31.57 ± 3.10 | 371.17± 0.91 | 15.40 | 114.34 ± 3.20* | 160.00 ± 9.43 | 137.50 ± 3.54* | 36.43 ± 1.37 | 1.38 ± 0.20 | 1.18 ± 0.00 | 2.98 ± 0.02 | 9.40 ± 0.03 |
| **Tomebamba** | 7.54 ± 0.02 | 104.83 ± 0.12 | 6.85 ± 0.13 | 2.48 ± 0.18 | 304.50 ± 2.62 | 15.20 | 94.74 ± 2.14* | 95.00 ± 7.07 | 92.50 ± 10.61 | 3.20 ± 0.67 | 0.09 ± 0.00 | 0.42 ± 0.03 | 0.14 ± 0.00 | 5.50 ± 0.09 |
| **Zamora** | 7.00 ± 0.02 | 101.80 ± 0.44 | 6.24 ± 0.17 | 5.71 ± 0.32 | 288.53 ± 1.95 | 16.00 | 349.73 ± 2.14* | 867.50 ± 24.75 | 697.50 ± 24.75* | 5.75 ± 0.83 | 0.47 ± 0.02 | 0.42 ± 0.01 | 0.34 ± 0.00 | 3.27 ± 0.26 |
| **Aguarico** | 7.15 ± 0.13 | 57.01 ± 2.69 | 7.90 ± 0.08 | 82.33 ± 3.06 | 282.60 ± 5.83 | 19.30 | 24.83 ± 2.18 | 242.50 ± 24.75 | 92.50 ± 3.54 | 8.73 ± 1.53 | 0.15 ± 0.01 | 0.49 ± 0.06 | 0.98 ± 0.00 | 6.25 ± 0.23 |
| **Coca** | 7.22 ± 0.09 | 77.33 ± 0.08 | 7.27 ± 0.13 | 105.00 ± 4.00 | 412.77 ± 0.80 | 18.90 | 69.63 ± 6.72* | 225.00 ± 42.43 | 182.50 ± 10.61* | 2.17 ± 0.10 | 0.08 ± 0.00 | 0.32 ± 0.03 | 0.18 ± 0.00 | 8.28 ± 0.03 |
| **Napo** | 6.89 ± 0.06 | 365.83 ± 0.75 | 7.64 ± 0.22 | 124.67 ± 4.04 | 62.44 ± 0.02 | 22.00 | 19.72 ± 0.00 | 592.50 ± 17.68 | 65 ± 7.07 | 1.39 ± 0.13 | 0.11 ± 0.01 | 0.30 ± 0.01 | 0.91 ± 0.00 | 5.87 ± 0.06 |
| **Pastaza** | 6.99 ± 0.04 | 48.37 ± 0.51 | 6.08 ± 0.34 | 2.50 ± 0.53 | 343.27 ± 25.37 | 23.40 | 26.85 ± 10.08 | 80.00 ± 28.28 | 237.50 ± 3.54* | 3.72 ± 0.10 | 0.18 ± 0.01 | 0.48 ± 0.09 | 0.04 ± 0.00 | 3.50 ± 0.09 |

^a^ Table 2. Quality criteria acceptable for the preservation of aquatic and wildlife in freshwaters, cold or warm, and marine waters and estuaries. Texto Unificado Legislación Secundaria del Medio Ambiente (TULSMA), Book VI, Annex I (Ministry of Environment of Ecuador (MAE) 2015a)

^b^ Table 9. Discharge limits to a freshwater body. TULSMA, Book VI, Annex I (Ministry of Environment of Ecuador (MAE) 2015a)

MCL: Maximum Contaminant Level; *Values that exceed the quality criteria; N/A: not available. The reported values were obtained by triplicate measurements of each analyzed river sample.
