# Supplementary material for: Determining the microbial and chemical contamination in Ecuador’s main rivers
Source: Sci Rep. 2021 Sep 3;11:17640. doi: 10.1038/s41598-021-96926-z (PMC8531378; doi:10.1038/s41598-021-96926-z)
Supplement: Supplementary file 5 — Supplementary Information 5. [file 41598_2021_96926_MOESM5_ESM.docx]

Manuscript title: **Determining the microbial and chemical contamination in Ecuador’s main rivers**

Authors: Dayana Vinueza, Valeria Ochoa- Herrera, Laurence Maurice, Esteban Tamayo, Lorena Mejía, Eduardo Tejera, and António Machado

**Supplementary Information**

**Table S3 – Average and standard deviation values of trace metals and major elements from the twelve rivers analyzed in this study.**

|  | **Trace metals** | | | | | | **Major elements** | | | | | | |
| --- | --- | --- | --- | --- | --- | --- | --- | --- | --- | --- | --- | --- | --- |
| **River** | **Copper**  **(µg L^-1^)** | **Chromium**  **(µg L^-1^)** | **Manganese**  **(µg L^-1^)** | **Lead**  **(µg L^-1^)** | **Lithium**  **(µg L^-1^)** | **Zinc**  **(µg L^-1^)** | **Aluminum**  **(mg L^-1^)** | **Iron**  **(mg L^-1^)** | **Magnesium**  **(mg L^-1^)** | **Calcium**  **(mg L^-1^)** | **Sodium**  **(mg L^-1^)** | **Potassium**  **(mg L^-1^)** |  |
| **MCL** | **5^a^** | **32^a^** | **100^a^** | **1^a^** | **2500^b^** | **30ª** | **0.1^a^** | **0.3^a^** | **N/A** | **N/A** | **N/A** | **N/A** |  |
| **Esmeraldas** | < LOQ | 1.52 ± 0.05 | 1.14 ± 0.05 | < LOQ | 11.42 ± 0.14 | 29.50 ± 0.14 | 22.26* ± 0.10 | 0.04 ± 0.00 | 3.20 ± 0.01 | 8.12 ± 0.58 | < LOQ | 1.78 ± 0.24 |  |
| **Toachi** | N/A | N/A | N/A | N/A | N/A | N/A | N/A | N/A | N/A | N/A | N/A | N/A |  |
| **Chone** | < LOQ | 1.49 ± 0.02 | 15.63 ± 0.46 | 10.12* ± 0.70 | 9.64 ± 0.21 | 88.91* ± 0.97 | 22.45* ± 0.19 | 0.12 ± 0.00 | 2.27 ± 0.04 | 2.87 ± 0.06 | 4.85 ± 0.15 | 2.92 ± 0.03 |  |
| **Guayas** | 154.67* ± 2.20 | 4.86 ± 0.02 | 65.08 ± 0.48 | 10.73* ± 0.09 | 17.39 ± 0.22 | 93.58* ± 0.12 | 30.80* ± 0.37 | 6.84* ± 0.03 | 64.18 ± 0.22 | 33.76 ± 0.36 | 578.82 ± 2.12 | 21.43 ± 0.27 |  |
| **Machángara** | 38.95*± 1.46 | 5.52 ± 0.29 | 162.37* ± 6.55 | 10.82* ± 0.06 | 13.91 ± 0.48 | 41.62* ± 1.05 | 22.17* ± 0.01 | 0.01 ± 0.00 | 1.47 ± 0.16 | 2.20 ± 0.49 | 31.76 ± 1.03 | 1.73 ± 0.46 |  |
| **Guayllabamba** | 10.17* ± 0.00 | 2.86 ± 0.00 | 75.50 ± 0.00 | < LOQ | 17.38 ± 0.64 | 104.84* ± 0.00 | 0.49* ± 0.00 | 0.46* ± 0.00 | 13.33 ± 0.43 | 17.86 ± 2.13 | 30.71 ± 1.43 | N/A |  |
| **Tomebamba** | < LOQ | 1.54 ± 0.20 | 8.75 ± 0.10 | < LOQ | 3.69 ± 0.01 | 127.02* ± 1.01 | 22.44* ± 0.07 | 0.10 ± 0.00 | 2.08 ± 0.17 | 15.89 ± 0.24 | < LOQ | 1.76 ± 0.29 |  |
| **Zamora** | < LOQ | 1.80 ± 0.01 | 85.94 ± 3.36 | < LOQ | 4.83 ± 0.11 | 46.74* ± 1.89 | 22.25* ± 0.04 | 0.28 ± 0.01 | 15.63 ± 1.04 | 45.69 ± 3.14 | 33.03 ± 2.22 | 5.71 ± 0.08 |  |
| **Aguarico** | N/A | N/A | N/A | N/A | N/A | N/A | N/A | N/A | N/A | N/A | N/A | N/A |  |
| **Coca** | < LOQ | 12.93 ± 0.24 | 6.60 ± 0.07 | < LOQ | 7.25 ± 0.14 | 69.51* ± 1.29 | 22.11* ± 0.10 | 0.17 ± 0.01 | 16.21 ±0.26 | 16.32 ± 0.42 | 100.84 ± 2.19 | 5.76 ± 0.44 |  |
| **Napo** | < LOQ | < LOQ | < LOQ | < LOQ | 3.35 ± 0.04 | 45.72* ± 0.24 | 22.16* ± 0.13 | 0.02 ± 0.00 | 13.11 ± 0.70 | 23.60 ± 1.94 | 51.07 ± 3.54 | 12.62 ± 0.54 |  |
| **Pastaza** | N/A | N/A | N/A | N/A | N/A | N/A | N/A | N/A | N/A | N/A | N/A | N/A |  |

^a^ Table 2. Quality criteria acceptable for the preservation of aquatic and wildlife in freshwaters, cold or warm, and marine waters and estuaries. Texto Unificado Legislación Secundaria del Medio Ambiente (TULSMA), Book VI, Annex I (Ministry of Environment of Ecuador (MAE) 2015a)

^b^ Table 3. Quality criteria for water for agricultural irrigation. TULSMA, Book VI, Annex I (Ministry of Environment of Ecuador (MAE) 2015c)

MCL: Maximum Contaminant Level; * Values that exceed the quality criteria; <LOQ: below the limit of quantification; N/A: not available. The reported values were obtained by triplicate measurements of each analyzed river sample.
