# Supplementary material for: Determining the microbial and chemical contamination in Ecuador’s main rivers
Source: Sci Rep. 2021 Sep 3;11:17640. doi: 10.1038/s41598-021-96926-z (PMC8531378; doi:10.1038/s41598-021-96926-z)
Supplement: Supplementary file 6 — Supplementary Information 6. [file 41598_2021_96926_MOESM6_ESM.docx]

Manuscript title: **Determining the microbial and chemical contamination in Ecuador’s main rivers**

Authors: Dayana Vinueza, Valeria Ochoa- Herrera, Laurence Maurice, Esteban Tamayo, Lorena Mejía, Eduardo Tejera, and António Machado

**Supplementary Information**

**Table S4 – Primers and PCR cycling parameters for the detection of various potential bacterial pathogens.**

| **Organism** | **Primer name** | **Primer sequence (5′–3′)** | **PCR cycling parameters** | **Gene (size [bp])** | **References** |
| --- | --- | --- | --- | --- | --- |
| Universal | Forward: fDD2 | CCGGATCCGTCGACAGAGTTTGATCITGGCTCAG | 3 min at 94 °C; 35 cycles of 94 °C for 30 s, 54 °C for 30 s, 72°C for 1.5 min | *16S* rRNA (1,600) | 64  (Rawlings et al., 1999) |
|  | Reverse: rPP2 | CCAAGCTTCTAGACGGITACCTTGTTACGACTT |  |  |  |
| *Shigella* spp. | Forward: IpaH-F | CCTTGACCGCCTTTCCGATA | 2 min at 95 °C; 35 cycles of 94 °C for 1 min, 62 °C for 1 min, 72 °C for 2.5 min | Invasion plasmid antigen H (606) | 65  (Kong et al. 2002) |
|  | Reverse: IpaH-R | CAGCCACCCTCTGAGGTACT |  |  |  |
| *Legionella* spp. | Forward: JFP | AGGGTTGATAGGTTAAGAGC | 5 min at 95 °C; 40 cycles of 94 °C for 1 min, 57 °C for 1.5 min, 72 °C for 1 min | Attachment invasion locus gene (386) | 66  (Jonas et al. 1995) |
|  | Reverse: JRP | CCAACAGCTAGTTGACATCG |  |  |  |
| *Salmonella* spp. | Forward: IpaB-F | GGACTTTTTAAAAGCGGCGG | 2 min at 95 °C; 35 cycles of 94 °C for 1 min, 62 °C for 1 min, 72 °C for 2.5 min | Invasion plasmid antigen B (314) | 65  (Kong et al. 2002) |
|  | Reverse: IpaB-R | GCCTCTCCCAGAGCCGTCTGG |  |  |  |
| *Pseudomonas* spp. | Forward: PA-GS-F | GACGGGTGAGTAATGCCTA | 2 min at 95 °C; 35 cycles of 94 °C for 20 s, 54 °C for 20 s, 72 °C for 40 s | *16S* rRNA (618) | 67  (Spilker et al. 2004) |
|  | Reverse: PA-GS-R | CACTGGTGTTCCTTCCTATA |  |  |  |
| *Campylobacter* spp. | Forward: IC-F | CTAGAGTACAAACTAATAAGTCTC | 3 min at 95 °C; 30 cycles of 94 °C for 45 s, 52 °C for 45 s, 72 °C for 45 s | Flanking regions of *ITS* gene (700) | 68  (Khan and Edge, 2007) |
|  | Reverse: IC-R | ATTCTAAAACGCATCACTTCCTTG |  |  |  |

(Dobrowsky et al. 2014)
