# Supplementary material for: Determining the microbial and chemical contamination in Ecuador’s main rivers
Source: Sci Rep. 2021 Sep 3;11:17640. doi: 10.1038/s41598-021-96926-z (PMC8531378; doi:10.1038/s41598-021-96926-z)
Supplement: Supplementary file 7 — Supplementary Information 7. [file 41598_2021_96926_MOESM7_ESM.docx]

Manuscript title: **Determining the microbial and chemical contamination in Ecuador’s main rivers**

Authors: Dayana Vinueza, Valeria Ochoa- Herrera, Laurence Maurice, Esteban Tamayo, Lorena Mejía, Eduardo Tejera, and António Machado

**Supplementary Information**

**Table S5 – Primers and PCR cycling parameters for the detection and identification of *E. coli* pathotypes accordingly to Toma et al. (2003) ^69^.**

| **Organism** | **Primer name** | **Primer sequence (5′–3′)** | **PCR cycling parameters** | ***Gene* (size [bp])** |
| --- | --- | --- | --- | --- |
| EAEC | Forward: AggRKs1 | GTATACACAAAAGAAGGAAGC | 2 min at 95 °C; 35 cycles of 95 °C for 1 min, 54 °C for 1 min, 72 °C for 1 min | *aggR* (254) |
|  | Reverse: AggRkas2 | ACAGAATCGTCAGCATCAGC |  |  |
| EHEC | Forward: VTcomU | GAGCGAAATAATTTATATGTG |  | *stx* (518) |
|  | Reverse: Vtcomd | TGATGATGGCAATTCAGTAT |  |  |
| EPEC | Forward: SK1 | CCCGAATTCGGCACAAGCATAAGC |  | *eae* (881) |
|  | Reverse: SK2 | CCCGGATCCGTCTCGCCAGTATTCG |  |  |
| EIEC | Forward: IpaIII | GTTCCTTGACCGCCTTTCCGATACCGTC |  | *ipaH* (619) |
|  | Reverse: IpaIV | GCCGGTCAGCCACCCTCTGAGAGTAC |  |  |
