# Supplementary material for: Determining the microbial and chemical contamination in Ecuador’s main rivers
Source: Sci Rep. 2021 Sep 3;11:17640. doi: 10.1038/s41598-021-96926-z (PMC8531378; doi:10.1038/s41598-021-96926-z)
Supplement: Supplementary file 8 — Supplementary Information 8. [file 41598_2021_96926_MOESM8_ESM.docx]

Manuscript title: **Determining the microbial and chemical contamination in Ecuador’s main rivers**

Authors: Dayana Vinueza, Valeria Ochoa- Herrera, Laurence Maurice, Esteban Tamayo, Lorena Mejía, Eduardo Tejera, and António Machado

**Supplementary Information**

**Table S6** **– Recovery percentage, reproducibility percentage, accuracy percentage, detection, and quantification limits obtained from metal analysis employing the ICP-OES.**

| Elements  Parameters | | Al | Ba | Cd | Cr | Cu | Fe | Pb | Mn | Ni | V | Zn | Co | Ca | Mg | Na |
| --- | --- | --- | --- | --- | --- | --- | --- | --- | --- | --- | --- | --- | --- | --- | --- | --- |
| Recovery (%) | Run1 | 90.67 | 95.40 | 96.19 | 97.24 | 96.72 | 93.07 | 105.81 | 96.67 | 89.43 | 101.41 | 94.75 | - | 105.42 | 93.43 | 100.38 |
|  |  |  |  |  |  |  |  |  |  |  |  |  |  |  |  |  |
|  | Run 2 | - | - | 112.08 | 105.21 | 101.70 | - | - | 93.27 | 103.97 | 100.53 | 100.92 | 98.98 | 91.81 | 118.53 | 96.04 |
| Limit of detection (LOD) (µg L^-1^) | Run1 | 1.92 | 4.09 | 0.16 | 0.29 | 1.23 | 0.78 | 3.24 | 0.18 | 1.00 | 2.03 | 0.42 | - | 0.20 | 0.06 | 0.04 |
|  | Run 2 | - | - | 0.24 | 0.40 | 2.50 | - | - | 0.25 | 0.83 | 0.63 | 0.65 | 0.51 | 0.02 | 0.06 | 0.09 |
| Limit of quantification (LOQ)  (µg L^-1^) | Run 1 | 6.39 | 13.62 | 0.52 | 0.97 | 4.11 | 2.61 | 10.12 | 0.60 | 3.34 | 6.77 | 1.40 | - | 0.66 | 0.20 | 0.14 |
|  | Run 2 | - | - | 0.80 | 1.33 | 8.32 | - | - | 0.83 | 2.77 | 2.09 | 2.16 | 1.71 | 0.06 | 0.021 | 0.31 |
| Accuracy (%) | Run 1 | 0.19 | 2.95 | 4.89 | 3.55 | 1.61 | 0.34 | 13.09 | 2.44 | 2.55 | 6.75 | 2.58 | - | 0.07 | 2.97 | 0.78 |
|  | Run 2 | - | - | 3.92 | 4.12 | 4.91 | - | - | 4.72 | 4.08 | 5.47 | 3.87 | 4.58 | 0.00 | 5.30 | 5.73 |
|  | Average | 0.19 | 2.95 | 4.40 | 3.83 | 3.26 | 0.34 | 13.09 | 3.58 | 3.32 | 6.11 | 3.23 | 4.58 | 0.03 | 4.13 | 3.25 |
|  | σ | - | - | 0.69 | 0.40 | 2.34 | - | - | 1.61 | 1.08 | 0.90 | 0.91 | - | 0.03 | 1.16 | 2.47 |
| Reproducibility | Run 1 | 0.09 | 0.05 | 0.04 | 0.03 | 0.03 | 0.07 | 0.06 | 0.03 | 0.11 | 0.01 | 0.05 | - | 0.05 | -0.07 | 0.00 |
|  | Run 2 | - | - | 0.12 | 0.05 | 0.02 | - | - | 0.07 | 0.04 | 0.01 | 0.01 | 0.01 | -0.08 | 0.19 | -0.04 |
|  | Average | 0.09 | 0.05 | 0.08 | 0.04 | 0.02 | 0.07 | 0.06 | 0.05 | 0.07 | 0.01 | 0.03 | 0.01 | -0.01 | 0.06 | -0.02 |
|  | σ | - | - | 0.06 | 0.02 | 0.01 | - | - | 0.02 | 0.05 | 0.01 | 0.03 | - | 0.06 | 0.13 | 0.02 |
| CRM 1640a (µg L^-1^) | | 53.00 | 151.80 | 3.99 | 40.54 | 85.75 | 36.80 | 12.10 | 40.39 | 25.32 | 15.05 | 55.64 | 20.34 | 5.61 | 1.05 | 3.13 |

Legend: Values obtained with recovery percentages outside of the acceptable range (between 80 and 100%) were replaced with (-) signs.
